# A new Kayvirus vB_SauM-MUHD-1 combats Methicillin-resistant *Staphylococcus aureus* wound infections

**DOI:** 10.1007/s00253-026-13898-8

**Published:** 2026-06-26

**Authors:** Maha Kabil, Noha T. Abou El-Khier, Wafaa Mowafy, Abeer M. Abd El-Aziz, Mohamed Abdelmoteleb

**Affiliations:** 1 Department of Medical Microbiology & Immunology, Faculty of Medicine, Mansoura University, Mansoura, Egypt; 2 Department of Microbiology and Immunology, Faculty of Pharmacy, Mansoura University, Mansoura, Egypt; 3 Botany Department, Faculty of Science, Mansoura University, Mansoura, Egypt

**Keywords:** MRSA, Wound infection, Phage therapy, Phage–antibiotic synergy, In vivo murine model

## Abstract

**Abstract:**

Methicillin-resistant *Staphylococcus aureus* (MRSA), a leading cause of chronic and post-surgical wound infections, is a hard-to-treat pathogen. In this study, we isolated and characterized a lytic MRSA bacteriophage, vB_SauM-MUHD-1, and evaluated its therapeutic potential in a murine wound infection model. Among 107 clinical wound samples, MRSA was reported in 48.6% of cases. Phage vB_SauM-MUHD-1, isolated from sewage, demonstrated lytic activity against 70.6% of the tested MRSA isolates. The phage exhibited efficient replication kinetics and remained stable under physiologically relevant conditions. Whole-genome sequencing identified a ~ 134 kb dsDNA genome (~ 30.45% GC) lacking detectable lysogeny-associated, virulence, or antimicrobial-resistance genes. In a BALB/c excisional wound model infected with MRSA, topical phage treatment significantly reduced bacterial burden, accelerated wound closure, and improved clinical severity scores compared to untreated controls, performing comparably to phage-linezolid combination therapy and outperforming linezolid monotherapy in bacterial clearance. These findings support that our phage vB_SauM-MUHD-1 has potential for treating MRSA-infected wounds and should be further investigated for efficacy in more challenging chronic or biofilm-rich wound environments.

**Key Points:**

• *This study provides a newly kayvirus, strictly lytic anti-MRSA phage vB_SauM-MUHD-1.*

• *Phage exhibited favorable replication kinetics, physical stability and genomic safety.*

• *Topical phage therapy reduced bacterial burden and accelerated wound healing.*

**Supplementary Information:**

The online version contains supplementary material available at 10.1007/s00253-026-13898-8.

## Introduction

*Staphylococcus aureus* (*S. aureus*) is a Gram-positive, opportunistic pathogen that causes a variety of infections in both hospital- and community-acquired infections, such as skin and soft tissue infections (SSTIs), osteomyelitis, pneumonia, surgical site infections, and bloodstream infections, and continues to be a significant source of morbidity and mortality globally (Kluytmans and Wertheim 2005; Tong et al. 2015). The rise and worldwide burden of MRSA, caused by the acquisition of the *mecA* gene that encodes the modified penicillin-binding protein PBP2a, has made the majority of β-lactam antibiotics ineffective and greatly restricted treatment choices (Chambers and Deleo 2009). MRSA is closely linked to persistent and challenging wound infections, burn injuries, and diabetic foot ulcers, where rates of mortality and complications related to infections continue to be significant (Kaushik et al. 2024; Rasmi et al. 2022). As a result, the World Health Organization has designated MRSA as a critical pathogen that necessitates immediate development of alternative therapeutic approaches (Mendelson and Matsoso 2015; WHO bacterial priority pathogens list 2024).

MRSA burden is complicated by its ability to create biofilms on injured tissue and medical equipment, along with its potential to gain extra resistance factors to macrolides, fluoroquinolones, aminoglycosides, and even last-line drugs like vancomycin and linezolid (Li et al. 2025; Tong et al. 2015). In chronic wounds and those related to burns, the combination of biofilm development, inadequate tissue perfusion, and weakened host immunity results in ongoing infection, postponed healing, and recurrent treatment failure, even with intensive antibiotic treatment (Li et al. 2025; Peng et al. 2022). The ongoing excessive use and inappropriate application of antibiotics in healthcare and community environments speeds up the emergence of multidrug-resistant (MDR) strains. This underscores the critical necessity to explore new or additional antimicrobial approaches beyond traditional antibiotics (Murray et al. 2022; Resistance ICGoA 2019). Among various alternative interventions, bacteriophage (phage) therapy has appeared as a promising method for addressing MDR *S. aureus* infections. Lytic phages can specifically target and eliminate bacterial cells. They are able to attach to surface receptors, inject their genetic material, and initiate a lytic replication process. This process preserves the normal host microbiota (Kortright et al. 2019; Łubowska et al. 2019). Several *Staphylococcus* phages, particularly those related to the *Herelleviridae* family and Kayvirus genus, demonstrated strong lytic abilities against MRSA. They have a wide host range, and significant antibiofilm efficacy, making them promising good candidates for treatment development (Łubowska et al. 2019; Seth et al. 2013; Simmonds et al. 2024). However, like antibiotics, bacteria can develop resistance to certain phages by receptor modifications or various defense mechanisms. This shows the importance of using phage cocktails and strategic combinations to improve longevity and effectiveness (Burmeister and Turner 2020; Mangalea and Duerkop 2020).

An emerging promising approach is combining phages with standard antibiotics in an approach known as phage-antibiotic synergy (PAS). In this approach, both phages and antibiotics work together. The destruction of bacterial cells and biofilm by phages can improve the effectiveness of antibiotics. At the same time, antibiotics may prevent the appearance of phage-resistant strains and reduce bacterial levels to amounts that phages can manage effectively (Comeau et al. 2007; Seth et al. 2013; Tagliaferri et al. 2019). It is important to note that while phage-antibiotic synergy shows promise, challenges remain, including the possibility of antagonistic interactions, the development of resistant bacteria, and intricate pharmacokinetic obstacles. These include optimizing dosing regimens, understanding mechanisms of action, and ensuring the safety and efficacy of these combinations in clinical settings (Rhoads et al. 2009; Fish et al. 2016; Nir-Paz et al. 2019).

In this study, we isolated, characterized, and sequenced a strictly lytic MRSA-specific bacteriophage from the class *Caudoviricetes* and evaluated its therapeutic potential in combination with linezolid. Using a murine model of MRSA-induced skin excisional wound infection, we assessed the efficacy of phage monotherapy, linezolid monotherapy, and phage–linezolid combination therapy on bacterial clearance, wound healing, and clinical outcomes. The aim was to determine whether combining a well-characterized lytic phage with a clinically important last-line antibiotic could enhance treatment outcomes, and provide a rational basis for developing phage–antibiotic combination regimens for MRSA-associated wound infections.

## Materials and methods

### Study design

This study is a laboratory-based experimental investigation that incorporates clinical sampling to isolate MDR *S. aureus* isolates, followed by bacteriophage isolation, characterization, genomic analysis, and evaluation of therapeutic efficacy in an experimental animal wound infection model. Clinical wound samples were collected from Mansoura University Hospitals over 12 months (March 2021–February 2022).

Ethical approval for both the clinical and animal components of the study was granted by the Ethical Committee of the Faculty of Medicine, Mansoura University, Egypt (Approval No. MD.20.08.352), which is responsible for the oversight of human and animal research. Written informed consent was obtained from all participants in accordance with the Declaration of Helsinki. Animal experiments were conducted in compliance with institutional and international guidelines.

### Clinical samples and bacterial isolates

A total of 107 patients with clinically infected wounds (burns, diabetic foot ulcers, traumatic and surgical wounds) were enrolled. Demographic and clinical data (age, sex, wound type, duration, site, comorbidities, prior antibiotic use, and outcome) were retrieved from medical records. Inclusion criteria were moderate–severe, clinically infected wounds requiring microbiological investigation. Patients with superficial/non-infected wounds, incomplete data, or > 72 h of systemic antibiotics before sampling were excluded. Wound swabs were collected after removal of dressings and gentle cleansing with sterile saline using Levine’s technique (rotation over a 1 cm^2^ area with firm pressure) (Levine et al. 1976). Swabs were placed into Stuart’s transport medium and processed promptly at the Microbiology Diagnostic and Infection Control Unit.

### MRSA identification and characterization

Swabs were vortexed in sterile saline, serially diluted, and plated onto nutrient agar, blood agar, and mannitol salt agar (MSA). Plates were incubated aerobically at 37 °C for 24 h. Wounds with ≥ 1 × 10^5^ CFU/swab were considered infected. Colonies suggestive of *Staphylococcus aureus* were identified by Gram staining (Gram-positive cocci in clusters), β-hemolytic golden colonies on blood agar, and yellow, mannitol-fermenting colonies on MSA. Biochemical confirmation included catalase (3% H₂O₂), tube coagulase (rabbit plasma), and oxidase tests using standard procedures (Fernandes et al. 2021).

### Antimicrobial susceptibility testing of MRSA isolate

Antimicrobial susceptibility of MRSA isolates was determined by Kirby–Bauer disc diffusion on Mueller–Hinton agar following the Clinical and Laboratory Standards Institute (CLSI) guidelines (CLSI M100, 32nd edition, 2022). Resistance against Methicillin was screened and identified using cefoxitin as a surrogate marker, and multidrug resistance was defined as resistance to three or more antibiotic classes. Phenotypic results were confirmed using the VITEK® 2 Compact system, with *Staphylococcus aureus* ATCC 25923 used as a quality control strain (Magiorakos et al. 2012).

### Bacterial hunter strain growth curve

Among seven highly multidrug-resistant strains, MRSA-11 was selected for its strong phage amplification, and was chosen as the "hunter strain" for further phage work. The selected MDR *S. aureus* (hunter strain) isolate`s bacterial growth curve was determined photometrically and through viable counts, as mentioned by Balaure and Grumezescu (2020). A single colony was grown in nutrient broth. The bacterial growth was monitored by measuring optical density at 600 nm (OD₆₀₀) and detecting colony-forming units (CFU/mL) at regular time intervals. Growth phases were identified by comparing OD₆₀₀ readings with viable counts, and the resulting data were plotted against time using GraphPad Prism (version 10; GraphPad Software, San Diego, CA, USA) to create the growth curve (Balaure and Grumezescu 2020).

### Phage isolation and enrichment

Twelve sewage samples were collected following aseptic conditions from both healthcare-associated and urban drainage sites in Dakahlia Governorate during summer 2022. Samples were centrifuged at 4000 rpm, 20 min, 4 °C and filtered through 0.45 µm membranes. To enhance enrichment, 1 mL filtrate was mixed with 0.5 mL log-phase MRSA culture in 10 mL nutrient broth and incubated overnight at 37 °C while shaking. To obtain crude phage lysates, supernatants were recentrifuged and refiltered (Clokie and Kropinski 2009).

### Phage detection and purification

Enriched sewage filtrates were firstly screened for detection of any lytic activity against the selected MRSA “hunter” strain using broth lysis assays. If there was reduced turbidity relative to bacteria-only controls, this indicated potential phage-mediated lysis. Filtrates had an evidence of lytic activity were further evaluated by spot testing on MRSA soft-agar lawns to confirm bacterial growth inhibition (Muniesa et al. 2011).

Putative phage-containing samples were tested to double-layer agar plaque assays (Adams method) to find and count plaques and to calculate phage titers, shown as plaque-forming units per milliliter (PFU/mL). Well-isolated individual plaques that showed clear lytic characteristics were chosen and eluted into SM buffer. To guarantee clonality went through at least three consecutive rounds of single-plaque isolation (Kropinski et al. 2009).

High-titer phage lysates were obtained by propagating the purified clear phage stocks on the hunter strain, Then, they were precipitated with polyethylene glycol (PEG 8000) and NaCl. The concentrated lysates were centrifuged, resuspended in SM buffer, treated with chloroform to get rid of any remaining bacterial contamination, and filtered using 0.22 µm membranes (Panteleev et al. 2025). The final phage preparations were stored at 4 °C for short-term use and at − 80 °C in SM buffer supplemented with 15% glycerol for long-term preservation (Gill and Hyman 2010).

### Morphological characterization

For determination of plaque morphology of vB_SauM-MUHD-1, the double-layer agar method on lawns of the hunter strain was used. After 18–24 h incubation at 37 °C, Plaques were examined and characteristics including plaque clarity, diameter, margin definition, and halo formation were recorded.

Virion morphology was assessed by transmission electron microscopy (TEM). The purified high-titer phage suspensions were applied to carbon-coated copper grids, negatively stained with 2% (w/v) uranyl acetate, and investigated using a JEOL transmission electron microscope operated at 80 kV. Capsid diameter and tail length were measured from calibrated digital micrographs using image analysis software. At least 10 individual phage particles were analyzed to obtain representative morphometric data, and the results were expressed as mean ± standard deviation (SD), to support taxonomic classification based on virion structure (Curry et al. 2006).

### Biological characterization

The spot test was used firstly to assess the host range of vB_SauM-MUHD-1 against a panel of clinical MRSA isolates. As spot assays can overestimate susceptibility due to high local phage concentrations, they do not always indicate productive infection, host range was interpreted practically rather than strictly. Regarding the isolates that showed positive spot lysis, the efficiency of plating (EOP) was determined by double-layer plaque assays. The results were expressed relative to the propagating host strain, the Hunter strain. This method allowed us to distinguish between efficient, reduced, and nonproductive infections (Ferriol-González and Domingo-Calap 2021).

The assessment of the adsorption kinetics was conducted by combining phage suspensions with Hunter strain cultures in the mid-exponential growth phase and measuring the amount of unadsorbed phage particles at specific time intervals. For determination of Optimal multiplicity of infection (MOI), Hunter strain cultures were infected at varying MOIs and measuring resultant phage titers following incubation. One-step growth experiments were carried out at the selected MOI to determine eclipse period, latent period, and burst size, using standard protocols with periodic sampling and plaque enumeration (Abedon 2022).

### Physical characterization

The physical stability of vB_SauM-MUHD-1 was determined by incubating phage suspensions at different temperatures and pH values for 1 h, followed by determination of residual infectivity using plaque assays. Phage stability was expressed as the percentage of remaining titer relative to untreated controls (Jończyk-Matysiak et al. 2019).

### Phage genome sequencing and bioinformatic analysis

Genomic DNA was extracted from high-titer, purified lysates (~ 10⁹ PFU/mL) using phenol–chloroform extraction followed by column-based clean-up, and quantified spectrophotometrically. Libraries were prepared using the Nextera XT DNA Library Preparation Kit and sequenced on an Illumina MiSeq platform to generate paired-end reads (Baym et al. 2015). Reads were quality-checked and trimmed using FASTQC (Andrews 2010) and Trimmomatic (Bolger et al. 2014) respectively, and de novo assembly was performed with SPAdes (Bankevich et al. 2012). Quality of genome assembly was evaluated with QUAST (Gurevich et al. 2013).

Open reading frames were predicted using ORF-finder (Access date: July 2025; available at: www.ncbi.nlm.nih.gov/orffinder/) and functionally annotated via BLASTp searches against the NCBI non-redundant database (Altschul et al. 1997). Phage annotation was further compared to those of PATRIC (Davis et al. 2020) and RASTtk (Aziz et al. 2008). HHpred (Söding et al. 2005) and Interproscan (Jones et al. 2014) were implemented to detect protein homology and protein families. Genome maps were generated in SNAPGene (GSL Biotech; access date: July 2025; Available at: www.snapgene.com/).

Putative lysogeny, virulence, and antimicrobial resistance genes were screened using PhageLeads (Yukgehnaish et al. 2022) and curated databases to confirm suitability for therapeutic use (Arndt et al. 2016). DeepTMHMM was employed to identify transmembrane topology in the anticipated proteins. Transmembrane proteins are essential in viral pathogenicity and various replication stages, beginning with genome uncoating and concluding with viral release (Hallgren et al. 2022).

Intergenomic distances and taxonomic placement were assessed using VICTOR (Meier-Kolthoff and Göker 2017) and VIRIDIC (Moraru et al. 2020), and proteomic relationships were evaluated with ViPTree (Nishimura et al. 2017). In VICTOR, the genome-genome distances were calculated using the BLASTn top 100 matched phages. The Genome-BLAST Distance Phylogeny (GBDP) procedure was used for pairwise sequence alignments. Furthermore, intergenomic pairwise similarities were computed using the VIRDIC phylogeny, using default values of species (> 95%) and genus (> 70%). Additionally, a proteomic tree of vB_Ec_ZCEC14 was created using Viral Proteomic Tree (ViPTree) after tBLASTx (Altschul et al. 1990) genome-wide sequence alignments. Orthologous (signature) genes between vB_SauM-MUHD-1 and related phages identified from ViPTree were predicted using CoreGenes 0.5 (Turner et al. 2013). Conserved proteins were utilized to construct a phylogenetic tree in MEGA 11 (Tamura et al. 2021) employing top BLASTp matches from other phages. The CLUSTAL-W aligner was utilized to analyze the conserved proteins (Larkin et al. 2007), with a bootstrap of 1000 replicates and the default settings for the best Maximum Likelihood fit model.

### Murine MRSA-infected excisional wound model

#### Animals, infection, and treatment protocol

Forty-two female BALB/c mice (6–8 weeks, 18–22 g) were housed under standard conditions with free access to food and water. A full-thickness excisional wound infection model was used to establish localized MRSA infection. Mice were anesthetized using an intramuscular injection of ketamine (50 mg/kg) and xylazine (8 mg/kg) (NRC 2011; Flecknell 2015) The dorsal surface was shaved and disinfected with 10% povidone–iodine followed by 70% ethanol. Using a sterile circular stencil and scalpel, a 10 mm full-thickness excisional wound was created on the dorsal region of each animal under aseptic conditions. This model was selected to closely mimic infected cutaneous wounds and allow controlled assessment of bacterial burden and treatment efficacy. To minimize pain and distress, all animals received buprenorphine (0.05 mg/kg, subcutaneously) every 12 h for the first 48 h post-wounding. Purified, isolated bacteriophage was administered topically at a dose of 100 μL containing 1 × 10⁸ PFU once daily for five consecutive days. Prior to each application, the wound dressing was gently removed, and the phage suspension was applied directly onto the wound bed and granulation tissue. The wound was then covered with a sterile gauze pad and secured using a transparent Tegaderm dressing. Linezolid (Pfizer), reconstituted in sterile water for injection, was administered intraperitoneally at 25 mg/kg body weight once daily for five consecutive days (Galiano et al. 2004; Mendes et al. 2014).

Baseline swabs were obtained to exclude pre-existing *S. aureus* (Levine et al. 1976). The wounds of infected groups were inoculated with 100 µL of MRSA suspension (~ 3.1 × 10⁷ CFU/mL); control wounds received sterile saline (Shetru et al. 2021). Wounds were covered with sterile gauze and Tegaderm, and mice were housed individually. Mice were randomized into six groups (n = 7 per group): non-infected untreated control–negative control (G1), MRSA-infected saline-treated (G2) as a positive control, non-infected phage control (G3) as a safety control, MRSA + phage (G4), MRSA + linezolid (G5), and MRSA + phage + linezolid (G6) (Bozdogan et al. 2003; Louie et al. 2001). After 24 h to allow colonization, treatment was administered once daily for 5 days (Kifelew et al. 2020). Phage was applied topically as a high-titer suspension; linezolid was given intraperitoneally.

#### Outcome measures

Wound area was measured on days 0, 1, 3, 5, 7, 14 and 21 by tracing onto transparent sheets and calculating surface area; percentage wound closure was derived relative to day 0 (Galiano et al. 2004; Wang et al. 2013). Bacterial burden was assessed at the same time points by swabbing wounds, vortexing in broth, serially diluting, and plating onto MSA; CFU/mL were calculated from plates with 30–300 colonies (Bowler et al. 2001; Levine et al. 1976). Clinical condition (erythema, edema, exudate, fur condition, activity, and body weight) was monitored daily using a semi-quantitative scoring scale (0–3 per parameter) (Morton and Griffiths 1985; Stokes 2000). A composite score (0–21) was used to compare groups; animals reaching humane endpoints were euthanized.

### Statistical analysis

Statistical analysis was performed using GraphPad Prism (version 8.0). Prior to analysis, the normality of data distribution was assessed using the Shapiro–Wilk test implemented in RStudio software. Data are presented as mean ± SD. Normality testing confirmed that the data were approximately normally distributed, supporting the use of parametric statistical methods. Comparisons between groups at individual time points were performed using one-way ANOVA followed by Tukey’s post hoc test. As measurements were obtained repeatedly from the same animals over time, this approach was used for cross-sectional comparisons and does not account for within-subject correlations. A generalized estimating equation (GEE) model was employed to evaluate longitudinal changes in wound closure (%) and bacterial load across study groups over time. Group, time, and their interaction (group × time) were included in the model to assess differential treatment effects during follow-up. The negative control group (non-infected, untreated mice) served as the reference category. Results were expressed as β coefficients with 95% confidence intervals (CI), and statistical significance was set at p < 0.05.

## Results

### Hunter MRSA strain isolation

**Fig. 1:**
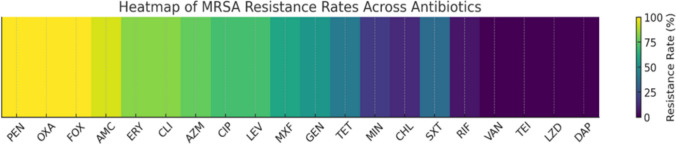
Antimicrobial resistance profile of MRSA isolates. Heatmap Heatmap visualization of resistance rates among 17 MRSA clinical isolates for 20 antibiotics. Highest resistance (≥ 94%) was observed for β-lactams, macrolides, and fluoroquinolones, while no resistance was detected to vancomycin, linezolid, teicoplanin, and daptomycin. Susceptibility testing was performed according to CLSI guidelines

Over the 12-month study period, wound swabs were collected from 107 patients with clinically infected wounds (63 males, 44 females; age range 21–67 years; mean ± SD = 44 ± 13.28). Patients were recruited from four Mansoura University–affiliated medical centers: Diabetic Foot Clinic (Specialized Medical Hospital), Surgical Ward (Mansoura University Hospital), Plastic Surgery and Burn Center, and Surgical Oncology Unit (OCMU), with diabetic foot ulcers representing the largest proportion of cases (52/107, 48.6%). Out of 107 wound swabs, 102 samples (95.3%) yielded positive bacterial growth. Single bacterial isolates were recovered from 85 samples (79.4%), whereas 17 samples (15.9%) showed polymicrobial growth (Supplementary material S1 and S2). Among the 35 confirmed *S. aureus* isolates, 17 (48.6%) were identified as methicillin-resistant based on cefoxitin disc diffusion testing, with confirmation by automated susceptibility testing (Fig. 1, Supplementary material S3). Seven highly multidrug-resistant strains were screened for their ability to support efficient phage replication. One single isolate which was identified as MRSA-11, showed consistently strong phage amplification and was selected as the "hunter strain" for further phage work. Growth curve analysis of MRSA-11 in nutrient broth at 37 °C identified a clear exponential phase between 2.0 and 5.5 h post-inoculation. It was noticed that the mid-exponential phase occurred at 4.5 h (OD₆₀₀ = 0.60), corresponding to ~ 3.1 × 10⁷ CFU/mL (Supplementary material S4), and this time point was used for subsequent phage infection assays.

### Lytic phage isolation

Twelve sewage samples collected from healthcare-associated and urban drainage sites were screened for bacteriophages active against clinical MRSA isolates using enrichment against the hunter strain. Initial broth lysis assays indicated putative phage activity in two enriched filtrates, as evidenced by reduced bacterial turbidity relative to bacteria-only controls. Subsequent spot testing confirmed lytic activity for these two samples (MUHD-1 and NSTD-3), whereas no lysis was observed for the remaining ten samples (Supplementary material S5).

For evaluation of phage infection productivity, double-layer agar plaque assays were used. Across experiments, well-defined and reproducible plaques were observed only from the MUHD-1 filtrate. In contrast, the plaques derived from the NSTD-3 filtrate were limited in number, poorly formed, and did not yield reproducible results upon retesting. As a result, only the phage obtained from MUHD-1 was chosen for further characterization. This phage produced clear, individual plaques on the lawns of the hunter strain and was named vB_SauM-MUHD-1 following the ICTV nomenclature guidelines (Supplementary material S5).

The first lysate of vB_SauM-MUHD-1 showed a titer of 2.4 × 10⁷ PFU/mL. The subsequent high-titer stocks were produced for additional representative plaque characteristics. Overall, these findings confirm the successful extraction of a strong lytic anti-MRSA bacteriophage from hospital sewage. This is also emphasizing the limited isolation of phages that consistently show lytic activity against clinical MRSA strains from environmental sources.

**Fig. 2:**
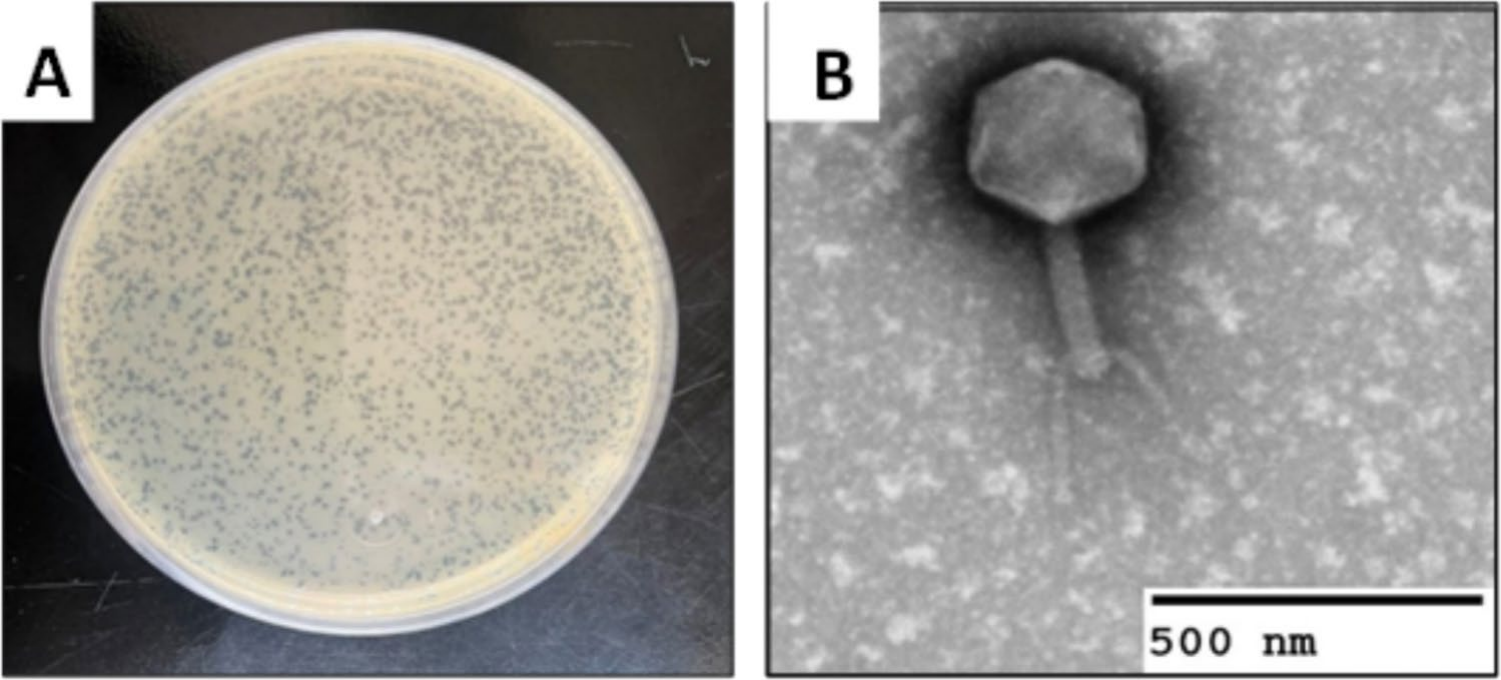
Morphological characterization of phage vB_SauM-MUHD-1. **A** Representative plaque morphology observed on MRSA-11 lawns following 18–24 h incubation at 37 °C, showing clear, circular plaques with sharp margins. **B** Transmission electron micrograph of negatively stained vB_SauM-MUHD-1 particles, revealing an icosahedral capsid and a long contractile tail characteristic of myophage morphology. Scale bar = 500 nm

Phage vB_SauM-MUHD-1 produced clear, circular plaques with well-defined edges after testing on MRSA-11 hunter strain lawns after being incubated for 18–24 h at 37 °C. The plaques exhibited uniform morphology, measuring an average of around 2.1 ± 0.4 mm in diameter, and showed stability across multiple passages, confirming a strictly lytic phenotype. Surrounding halos that would indicate polysaccharide depolymerase activity were not detected. Figure 2A illustrates the typical plaque morphology.

Transmission electron microscopy showed that vB_SauM-MUHD-1 has an icosahedral capsid and a long contractile tail. These features are typical of myophage morphology in the class *Caudoviricetes*. Measurements obtained from calibrated TEM micrographs of 11 individual virions showed that the isolated bacteriophage had a capsid diameter of 198 ± 12 nm, while the tail length ranged from 120 to 150 nm. These structural details confirm that vB_SauM-MUHD-1 belongs to the *Herelleviridae* family in the Kayvirus lineage. Representative TEM images are shown in Fig. 2B.

The lytic range of vB_SauM-MUHD-1 was evaluated against a group of 17 clinical MRSA strains. Preliminary spot assays showed that bacterial growth was inhibited in 12 out of 17 isolates (70.6%), while five isolates did not exhibit any noticeable lysis. Since spot assays may exaggerate susceptibility, the host range was considered operational rather than definitive, and further evaluation of productive infection was conducted through efficiency of plating (EOP). Among the 12 isolates that tested positive in the spot assays, seven had high EOP values (> 0.5), two had moderate EOP (0.1–0.5), and three demonstrated low EOP (< 0.1), reflecting varying phage–host compatibility among the clinical strains. Five isolates did not produce plaques and were identified as resistant. A summary of the lytic activity and EOP values for all examined MRSA strains can be found in Table 1.

**Table 1:** Lytic activity and efficiency of plating (EOP) of phage vB_SauM-MUHD-1 against MRSA isolates. The table summarizes phage source, resistance phenotype, and susceptibility patterns based on spot lysis and EOP values, classifying isolates into high, medium, low, or resistant categories

Isolate ID	Source	Resistance phenotype	Lytic Activity	EOP Value	EOP Category
MRSA-1	Diabetic foot ulcer	MDR *S. aureus*	Yes	0.87	High
MRSA-2	Diabetic foot ulcer	MDR *S. aureus*	Yes	0.74	High
MRSA-3	Diabetic foot ulcer	MDR *S. aureus*	Yes	0.59	High
MRSA-4	Diabetic foot ulcer	MDR *S. aureus*	Yes	0.12	Medium
MRSA-5	Diabetic foot ulcer	MDR *S. aureus*	Yes	0.65	High
MRSA-6	Diabetic foot ulcer	MDR *S. aureus*	No	–	Resistant
MRSA-7	Diabetic foot ulcer	MDR *S. aureus*	Yes	0.02	Low
MRSA-8	Diabetic foot ulcer	MDR *S. aureus*	Yes	0.93	High
MRSA-9	Surgical wound	MDR *S. aureus*	Yes	0.005	Low
MRSA-10	Surgical wound	MDR *S. aureus*	No	–	Resistant
MRSA-11	Diabetic foot ulcer	MDR *S. aureus*	Yes (Hunter strain)	1.00	High
MRSA-12	Surgical wound	MDR *S. aureus*	No	–	Resistant
MRSA-13	Burn wound	MDR *S. aureus*	Yes	0.43	Medium
MRSA-14	Burn wound	MDR *S. aureus*	No	–	Resistant
MRSA-15	Burn wound	MDR *S. aureus*	Yes	0.006	Low
MRSA-16	Bedsore wound	MDR *S. aureus*	No	–	Resistant
MRSA-17	Bedsore wound	MDR *S. aureus*	Yes	0.77	High

**Fig. 3:**
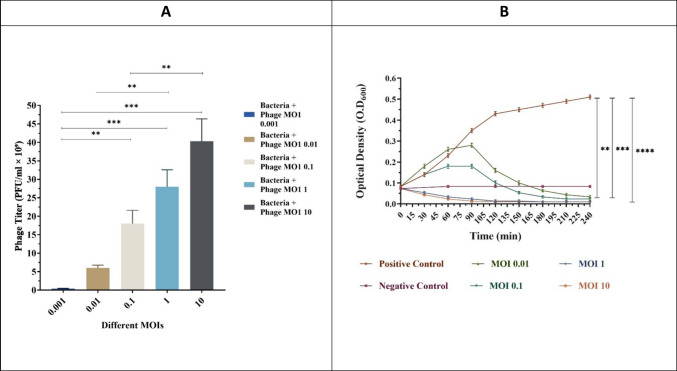
Replication efficiency of vB_SauM-MUHD-1 at different multiplicities of infection (MOIs). **A** Phage titers (PFU/mL) obtained following infection of MRSA-11 at MOIs ranging from 0.001 to 10. **B** Corresponding inhibition of MRSA-11 growth, measured as optical density reduction. Maximal phage yield and bacterial suppression were observed at an MOI of 10. Data represent mean ± SD of independent experiments

Experiments investigating the multiplicity-of-infection (MOI) using MRSA-11 revealed a clear increase in phage yield that depended on the MOI. The highest level of phage production occurred at an MOI of 10, resulting in a final titer of 3.9 × 10^1^⁰ PFU/mL and the most significant reduction in remaining bacterial density. Statistical evaluations indicated a notable impact of MOI on the final phage titers (one-way ANOVA, p < 0.0001). The relationships between phage yield and bacterial growth inhibition at different MOIs are illustrated in Fig. 3A and B, respectively.

**Fig. 4:**
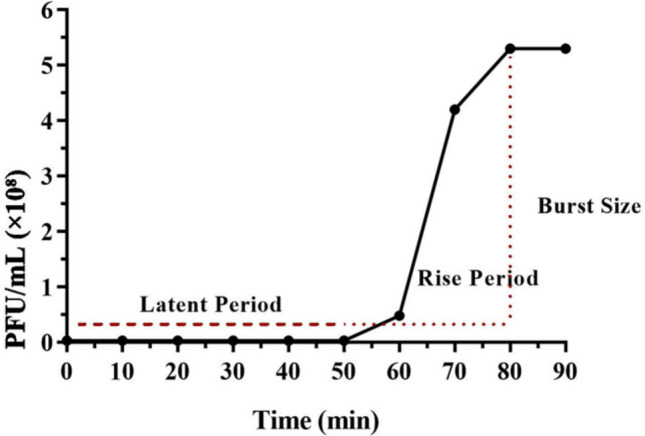
One-step growth curve of phage vB_SauM-MUHD-1 infecting MRSA-11 at MOI 0.01. Phage titers were determined at regular intervals following adsorption. The latent period (~ 55 min), and rise phase are indicated, with an estimated burst size of ~ 167 PFU per infected cell. Data represent mean ± SD of three independent assays

A one-step growth assessment indicated a latent phase of about 55 min. Following this, phage titers rose swiftly during the rise phase, reaching a plateau between 100 and 110 min after infection, which correlates to a burst size of roughly 167 PFU for each infected cell. The one-step growth curve along with the calculated replication parameters can be found in Fig. 4.

The stability of vB_SauM-MUHD-1 under different temperature and pH conditions was assessed to determine its suitability for therapeutic use. Phage infectivity largely remained intact after 1 h of incubation at temperatures up to 50 to 60 °C. However, there was a gradual loss of activity at higher temperatures, with complete inactivation observed at 80 °C.

**Fig. 5:**
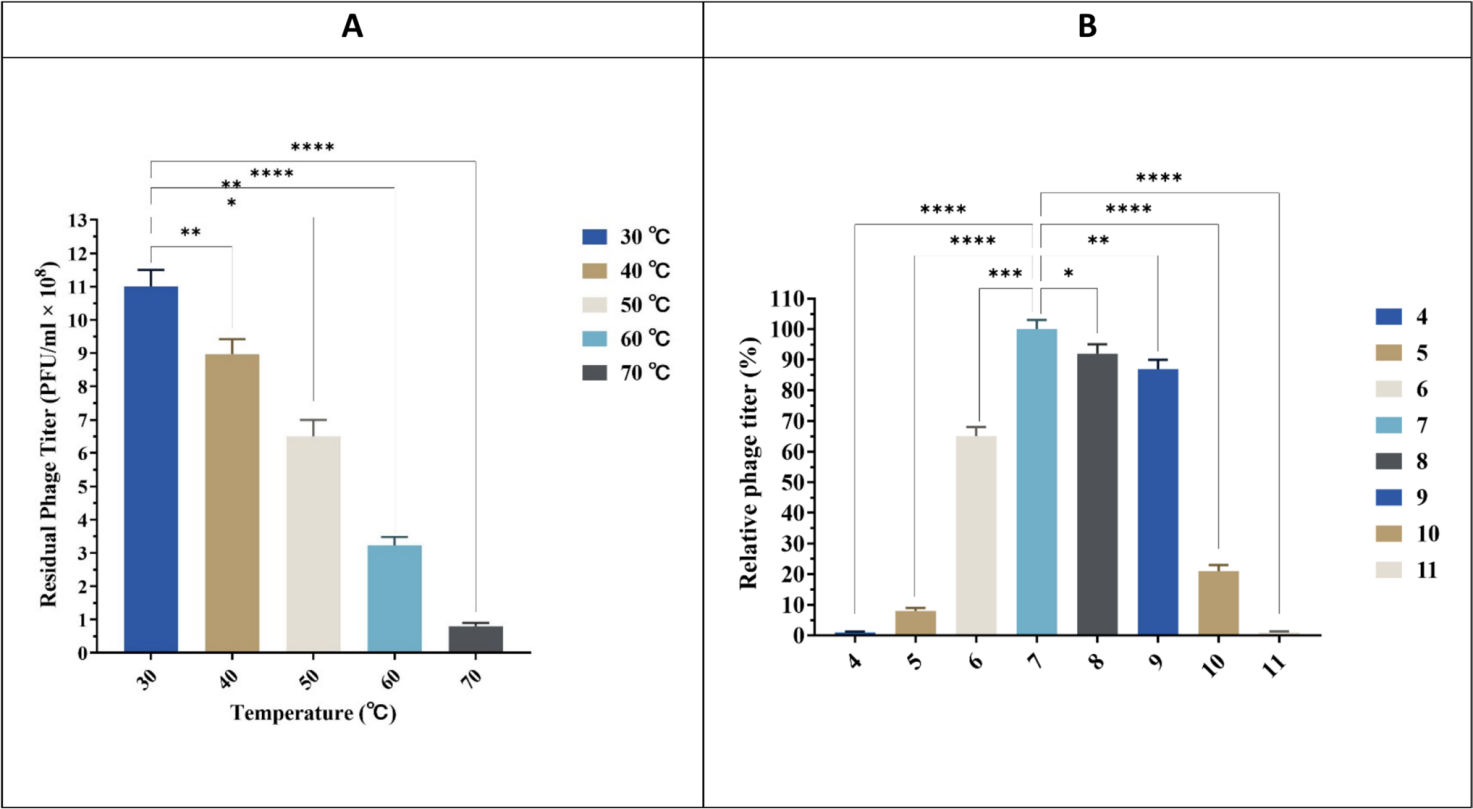
Physicochemical stability of phage vB_SauM-MUHD-1. **A** Thermal stability following 1 h incubation at the indicated temperatures. **B** pH stability following 1 h incubation across pH 4–11. Residual infectivity was quantified by plaque assay and expressed relative to untreated controls

pH stability tests showed that the phage was most stable between pH 7.0 and 8.0. It retained significant infectivity across a wider range from pH 6.0 to 9.0. Marked reductions in phage activity were observed under strongly acidic (≤ pH 5.0) or alkaline (≥ pH 10.0) conditions. Temperature- and pH-dependent stability profiles are summarized in Fig. 5A and B, respectively.

### Genomic characterization and Phylogenetic analysis

**Fig. 6:**
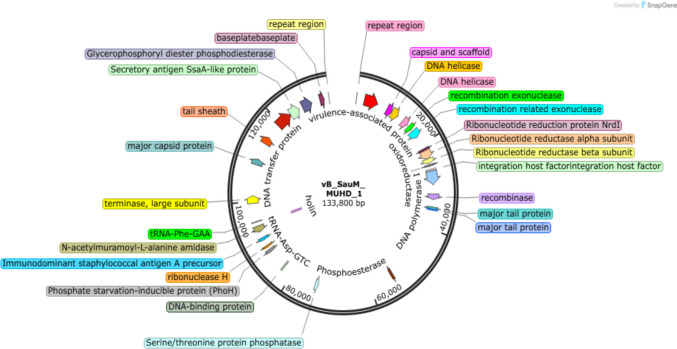
Genetic map of vB_SauM-MUHD-1 illustrating only proteins with assigned function

DNA sequencing of phage vB_SauM-MUHD-1 genome revealed a double-stranded DNA of ~ 134 kbp length. The phage genome was deposited into the NCBI GenBank (Accession No. PX848770). Genome assembly showed the following metrics: contigs no. 1; GC% 30.45; N50 133,800; L50 1; and 0 N’s per 100 kb. Two hundred and seven protein-coding genes were predicted through integration of different annotation platforms, among which functional proteins have allocated functions linked to phage structure, cell lysis and DNA replication/transcription/packaging/repair mechanisms (Supplementary material S6). Phage vB_SauM-MUHD-1 lacked any lysogenic genes, including integrases or transposases. On the plus strand, the phage vB_SauM-MUHD-1 contains one hundred fifty-four ORFs, whereas it possesses only sixty-five ORFs on the complementary strand. Genetic information is depicted on the genetic map, emphasizing functional genes as demonstrated in Fig. 6. Genomic examination revealed two repeats and two tRNAs (tRNA-Phe-GAA; tRNA-Asp-GTC).

Phage Leads explored the genome and didn’t report any genes associated with antibiotic resistance, lysogenic life cycle, or bacterial virulence. This suggests the relevance of vB_SauM-MUHD-1 for therapeutic uses. Transmembrane domains were identified in twenty-nine proteins through DeepTMHMM. Seven TMDs were only predicted in hypothetical protein (ORF 149) (Supplementary material S7). While three TMDs were recorded in three proteins (ORFs: 40, 87, and 177 (Supplementary material S7), two TMDs was recorded in fifteen proteins, and only one TMD in 10 proteins.

**Fig. 7:**
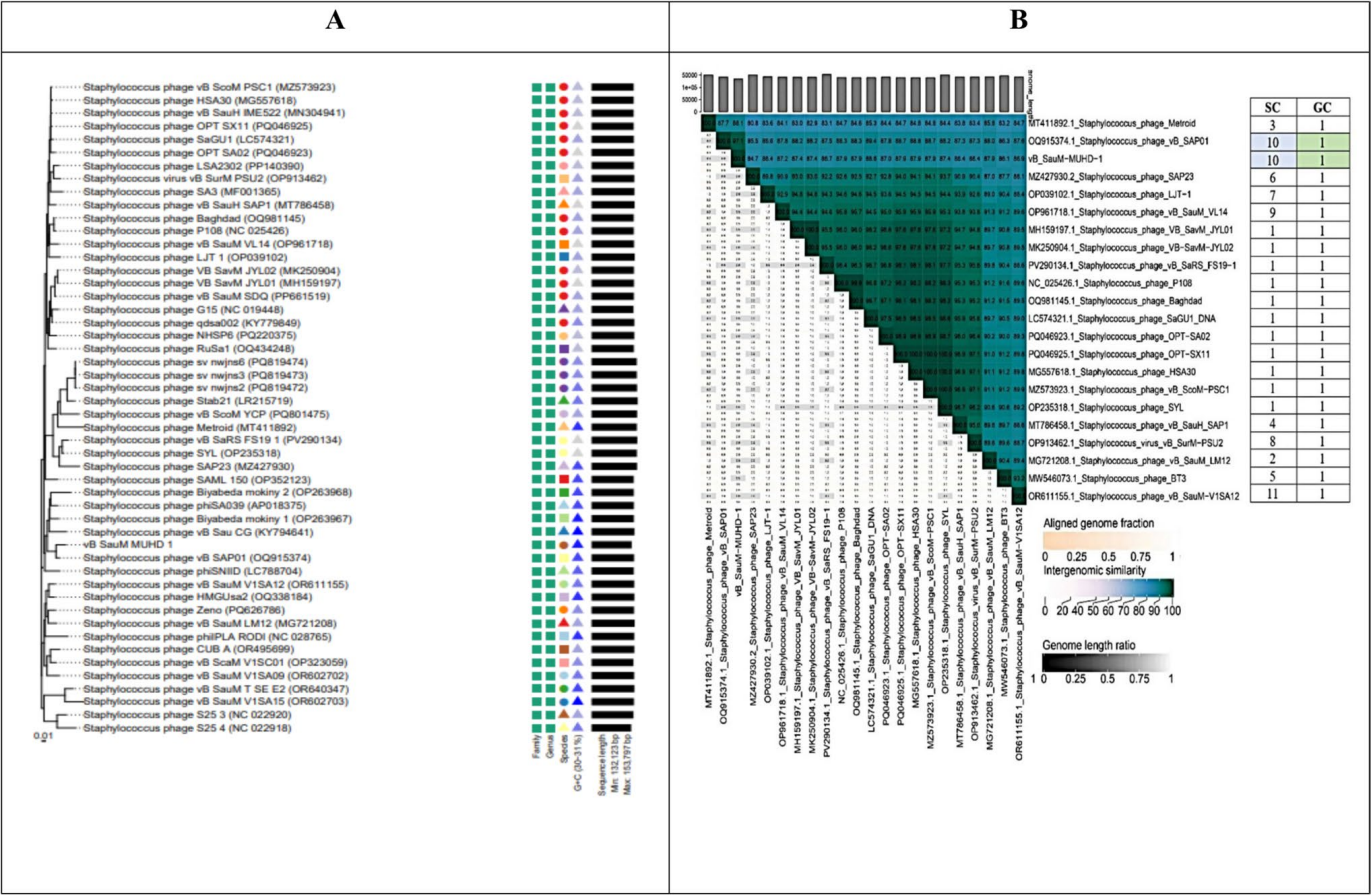
Phylogenetic analysis of vB_SauM-MUHD-1. **(A)** VICTOR phylogenetic tree of vB_SauM-MUHD-1 and top 50 BLASTn closely related phages; **(B)** VIRIDIC heatmap of phage vB_SauM-MUHD-1 and 21 BLASTn closely related phages

The phylogenetic analysis conducted out by VICTOR utilizing the phylogenomic GBDP platform has been derived from the D6 formula, resulting in 0% average support **(**Fig. 7A**)**. vB_SauM-MUHD-1 clustered with *Staphylococcus* phage vB_SAP01 in the same lineage. VIRDIC calculated the intergenomic similarities between vB_SauM-MUHD-1 and closely BLASTn related phages. *Staphylococcus* phages vB_SAP01 and SAP23 were clustered in the same species and genus thresholds with vB_SauM-MUHD-1 among the shortlisted 21 phages **(**Fig. 7B**)**.

**Fig. 8:**
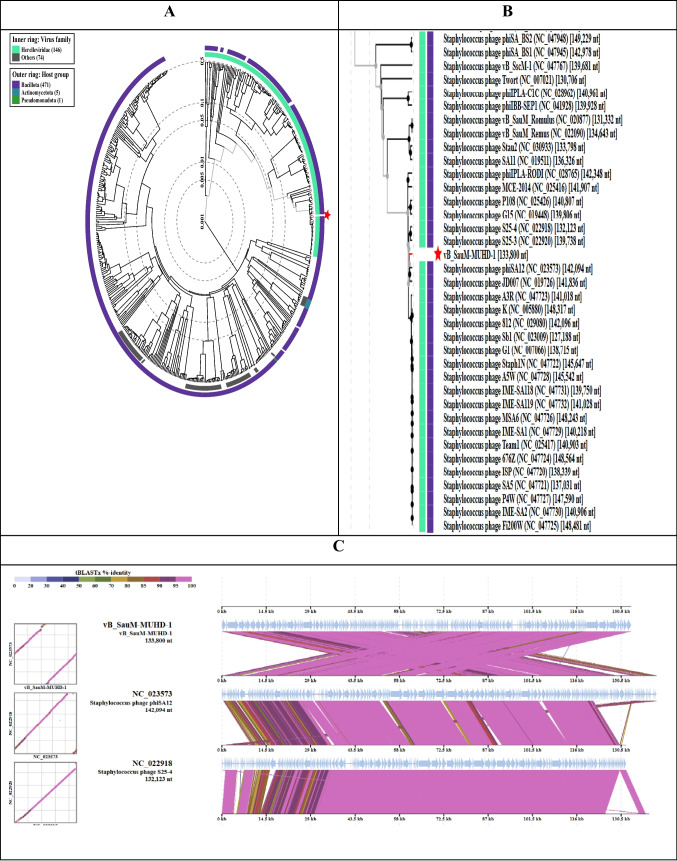
ViPTree proteomic tree of vB_SauM-MUHD-1 against RefSeq genomes of closely related phages. **A** Circular tree; **B** Rectangular tree illustrates a portion in the circular tree showing closely related phages. **(C)** Complete genome comparison of vB_SauM-MUHD-1 and ViPTree closely related phages

Furthermore, ViPTree proteomic tree confirmed that vB_SauM-MUHD-1 is a member of the *Herelleviridae* family which are infecting members of phylum *Firmicutes*, and grouped with other *Staphylococcus* phages **(**Fig. 8A, B**)**. As well, ViPTree inferred that vB_SauM-MUHD-1 is highly similar to *Staphylococcus* phages phiSA12 and S25-4. The entire genome of these closely related phages was compared and aligned with vB_SauM-MUHD-1, emphasizing the similarities and differences **(**Fig. 8C**)**. Coregenes analysis was conducted suing some chosen closely related *Staphylococcus* phages (vB_SAP01, SaGU1, HMGUsa2, vB_SauM_LM12, and P108). vB_SauM-MUHD-1 shared one hundred and eighty-eight orthologous genes. Three signature genes (PhoH family protein, large terminase subunit (Terl), and major capsid protein) were selected to perform sequence alignment and subsequent phylogenetic analysis in MEGA-11 (Supplementary material S8). In the three trees, signature genes of phage vB_SauM-MUHD-1 were clustered with those of other *Staphylococcus* phages. Following recent updates of International Committee on Taxonomy of Viruses (ICTV) 2025 release, genomic characteristics and host type of phage vB_SauM-MUHD-1 fit the family *Herelleviridae* of the class *Caudoviricetes*.

### In vivo efficacy of vB_SauM-MUHD-1 in a murine MRSA wound model

Baseline wound areas did not differ significantly among experimental groups. During the early post-infection period (Day 1), no significant differences in wound closure were observed (p > 0.05). From Day 3 onward, treatment-dependent differences became evident (p < 0.001). MRSA-infected untreated mice (G2) exhibited delayed and incomplete healing throughout the study period, with minimal wound closure observed during the first week and only partial closure by Day 21.

**Fig. 9:**
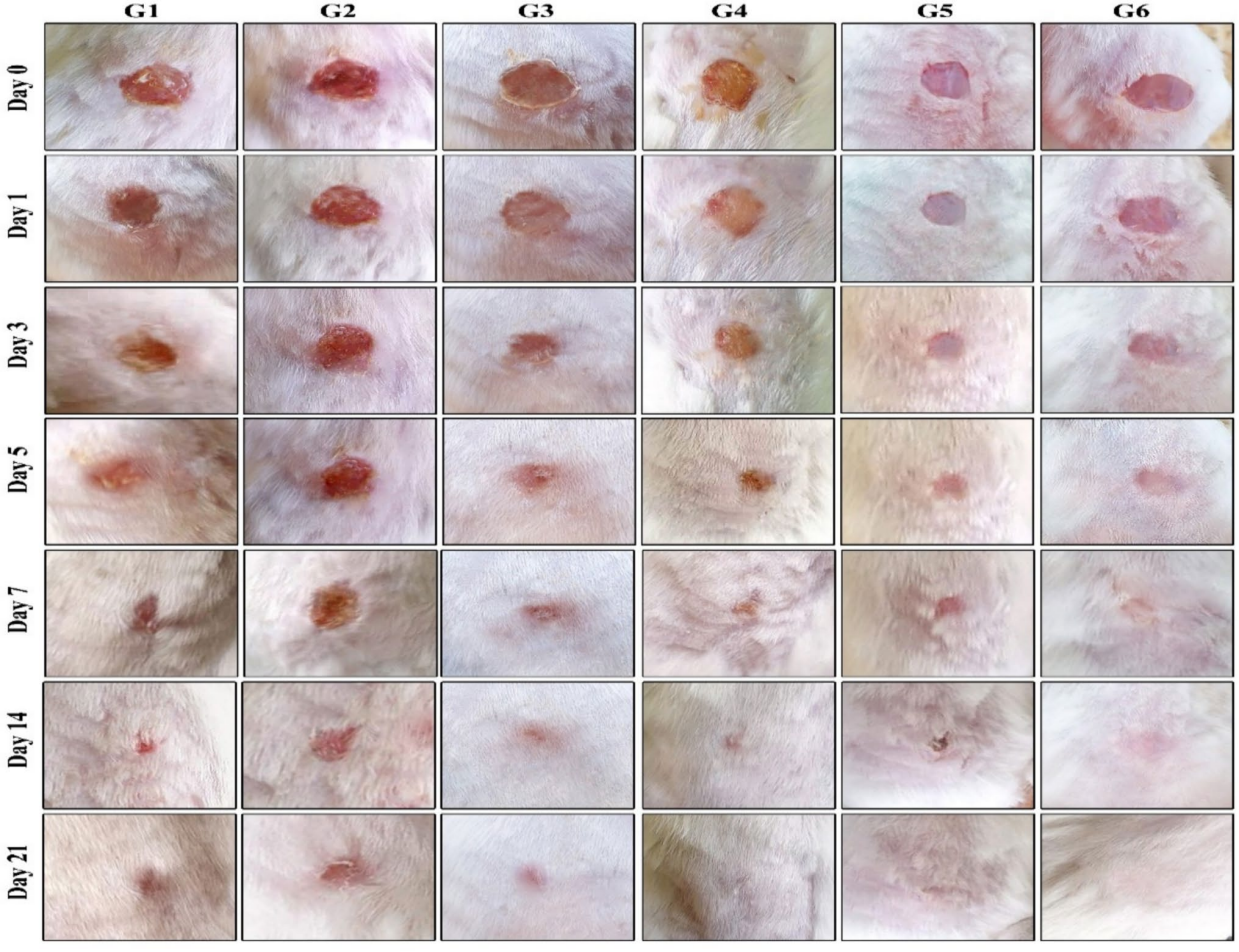
Time course of wound closure following MRSA infection and treatment. Percentage wound closure was assessed on Days 0, 1, 3, 5, 7, 14, and 21 post-infection. Phage-treated mice (G4) and phage–linezolid–treated mice (G6) demonstrated accelerated wound healing compared with untreated infected controls (G2), particularly from Day 3 onward. Linezolid monotherapy (G5) resulted in intermediate wound closure. Values represent mean ± SD (n = 7 per group)

**Fig. 10:**
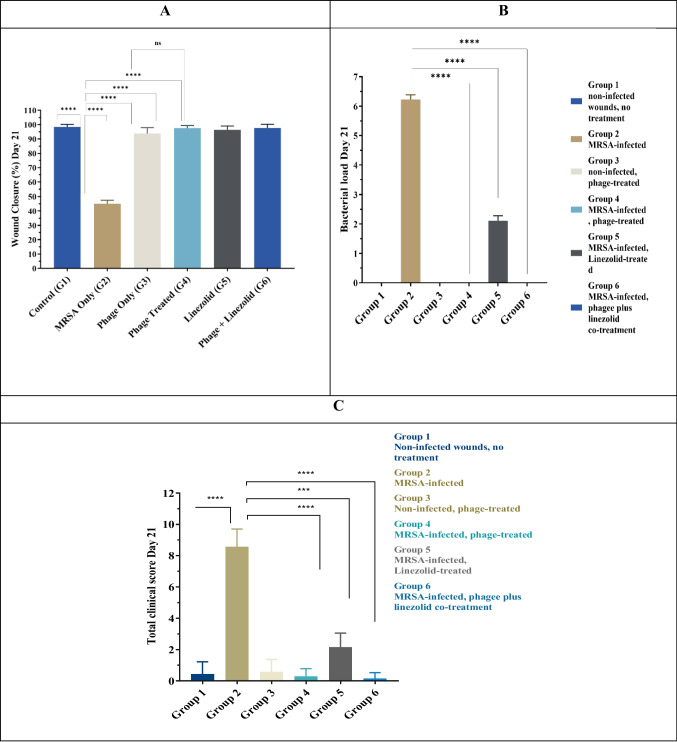
Wound closure (**A**), bacterial burden (**B**), and composite clinical severity scores (**C**) at Day 21 post-infection in the murine MRSA excisional wound model. Wound closure and bacterial burden data are presented as mean ± SD (n = 7 per group). Statistical significance was assessed using one-way ANOVA with appropriate post hoc testing. Composite clinical severity scores were represented as mean ± SD (n = 7 per group)

On the other hand, mice treated with phage monotherapy (G4) or a phage and linezolid combination therapy (G6) showed notable improvement in wound healing. By Day 5, both groups had much greater wound closure compared to untreated infected controls. By Days 7 to 14, wound closure surpassed 70 to 90%, getting close to that of non-infected controls. Monotherapy with linezolid (G5) resulted in better wound closure than untreated infected mice, but it consistently trailed behind the phage treatments at later evaluation points. There were no significant differences in wound closure between phage monotherapy and combination therapy at any of the evaluated time points across the experiments. Thus, our results demonstrate the superiority of phage monotherapy, which, along with known advantages of monotherapy, presents fewer pharmacokinetic challenges. Figures 9 and 10A show the results of wound closure and the healing profiles over time.

The quantification of bacterial load showed clear and significant differences among the treatment groups throughout the study. The untreated infected mice (G2) had high MRSA levels during the entire experiment, with only small decreases by Day 21. Mice treated with Linezolid (G5) had a steady decline in bacterial counts but still tested positive in cultures at later time points. On the other hand, mice treated with phage (G4) and those receiving a combination of phage and Linezolid (G6) experienced rapid and significant drops in bacterial load. By the seventh day, the bacterial levels in both groups receiving phage were markedly lower than those in the untreated and linezolid-only groups (p < 0.001). By Day 14, MRSA levels in both G4 and G6 had dropped below the detection threshold and continued to be undetectable until Day 21. There were no significant differences in bacterial clearance found between monotherapy with phage and the combination therapy. Figure 10B summarizes the comparisons of bacterial load.

Composite scores for clinical severity, which combine local wound inflammation with systemic health indicators, showed significant differences across groups throughout the 21-day observation period. Untreated infected mice (G2) displayed consistently high scores, indicating ongoing inflammation and a deteriorating overall condition. Linezolid alone (G5) led to some improvement but did not fully restore clinical scores to normal levels. Mice receiving phage therapy alone (G4) or a combination of phage and linezolid (G6) exhibited swift and continuous decreases in clinical severity, with scores nearing those of non-infected controls by the conclusion of the study. No notable differences were observed between the two phage treatment regimens at any assessment point. Clinical severity results on Day 21 are illustrated in Fig. 10C.

GEE analysis demonstrated a significant interaction between study group and time (p < 0.001), indicating that wound healing trajectories varied across groups. Compared with the negative control group (non-infected, untreated mice), both the bacterial control group (MRSA-infected) and the phage control group (non-infected, phage-treated) exhibited significantly less improvement in wound closure over time, particularly at later time points. In contrast, the phage-treated group, and the combination therapy group demonstrated significantly enhanced wound healing, with marked improvements observed during early and intermediate follow-up periods. These findings indicate that both treatment strategies achieved superior wound closure comparable to other groups (Supplementary material S9).

A significant group × time interaction was also observed for bacterial load (p < 0.001), reflecting differential bacterial reduction across treatment groups over time. Compared with the bacterial control group (MRSA-infected, treated with sterile saline), the phage-treated group, antibiotic-treated group, and combination therapy group showed significantly greater reductions in bacterial load, with the most pronounced effects observed in the phage-treated and combination therapy groups at later time points. Conversely, the phage control group showed no significant change in bacterial load throughout the study period, reflecting the absence of infection rather than a therapeutic effect (Supplementary material S10).

The data collected indicate that the topical application of vB_SauM-MUHD-1 enhanced wound healing, decreased bacterial load, and alleviated the severity of clinical disease in a mouse model of MRSA excisional wounds. Treatment with phage alone resulted in therapeutic effects similar to those seen with the combination of phage and linezolid and consistently surpassed the outcomes of linezolid treatment alone in both microbiological and clinical measures.

## Discussion

Environmental screening resulted in a small number of samples testing positive for phages, but only one of these exhibited consistent lytic activity and strong propagation against a clinical MRSA strain. This low recovery rate matches earlier findings showing that there is only a small portion of environmental bacteriophages have significant and clear therapeutic effects against some MDR clinical strains. The isolated phage, vB_SauM-MUHD-1, showed stable plaque characteristics, effective amplification, and a strictly lytic nature. These traits suggest its potential for further investigation (Altamirano and Barr 2019; Hyman 2019).

Morphological and biological assessments indicated that vB_SauM-MUHD-1 is a myophage that belongs to the *Herelleviridae* family, aligning with other extensively researched therapeutic phages targeting *Staphylococcus* (Hatfull and Hendrix 2011). Analysis of the host range showed lytic activity against around 70% of the clinical MRSA strains tested (Hyman and Abedon 2010). Notably, the interpretation of the host range was deemed operational rather than absolute, as spot assays were enhanced through efficiency-of-plating analysis to differentiate between productive infections and non-productive lysis. The observed variability in susceptibility mirrors the diversity of MRSA surface receptors and their defense strategies, which is typical of staphylococcal phages (Xia and Wolz 2014). This variability underlines the broader justification for developing future phage cocktails instead of depending on a single phage (Chan and Abedon 2012).

Replication kinetics showed a successful interaction between the phage and host, marked by a short latent period and a large burst size. These factors are helpful for therapy and improving outcomes at the infection site. furthermore, vB_SauM-MUHD-1 remained stable across relevant temperature and pH ranges, which is important for topical use in wound care (Ajuebor et al. 2018). These biological properties match those of other Kayvirus-lineage phages that have moved closer to clinical use (Glonti and Pirnay 2022).

The thorough genome analysis enables a comprehensive description of new phages and resolves the discrepancies from experimental findings. Whole-genome sequencing (~ 134 kb, GC 30.45%) revealed 207 ORFs organized into canonical structural, replication and lysis modules. Crucially, no genes encoding integrases, recombinases associated with lysogeny, known staphylococcal toxins, or antimicrobial resistance determinants were detected, aligning vB_SauM-MUHD-1 with current safety expectations for therapeutic phages and resembling established anti-MRSA Kayvirus phages such as K and Sb-1 (Philipson et al. 2018). A complete lysis cassette, including holin and amidase-type endolysin, and conserved structural and replication-associated genes further supported a strictly lytic lifestyle and robust bacteriolytic potential. Pinpointing transmembrane areas within the candidate proteins might indicate possible functions of these proteins. The DeepTMHMM tool detected several transmembrane domains, and the prediction of transmembrane topology relates to the protein's function, as it facilitates the entry of viral DNA into the infected bacterial cytoplasm by forming a channel in the bacterial cell membranes (Liu et al. 2023). Different methods were utilized for the phylogenetic analysis of vB_SauM-MUHD-1, and all these approaches yielded quite comparable outcomes. VIRIDIC classified vB_SauM-MUHD-1 together with other *Staphylococcus* phages (vB_SAP01 and SAP23). The VIRIDIC phylogeny is very dependable since it adheres to the ICTV guidelines in its methodology (Moraru et al 2020). Similarly, proteomic tree phylogeny showed that vB_SauM-MUHD-1 closely resembles *Staphylococcus* phages phiSA12 and S25-4. Proteomic trees offer understanding into the evolutionary history of phages (Hatfull and Hendrix 2011; Rohwer and Edwards 2002), supporting the investigation of more remote connections. The latest information from the International Committee on Taxonomy of Viruses (ICTV) indicates that the host characteristics and genomic features (GC content, genome size, number of coding sequences) of the family *Herelleviridae* in the class *Caudoviricetes* (Mäntynen et al. 2021), are similar to those of phage vB_AbaM_MU1.

To confirm these in vitro and genomic findings, an in vivo evaluation in a mouse model of excisional wound infection was used. Mice infected with untreated MRSA showed ongoing bacterial colonization, slow wound closure, and continued inflammation, reflecting major aspects of hard-to-treat wound infections (Morrison et al. 2024). Linezolid when used alone lowered the bacterial load and helped with healing, but it did not completely improve the outcomes. This aligns with the challenges of antibiotic treatment in long-standing wound infections (Uyttebroek et al. 2022).

On the other hand, applying of vB_SauM-MUHD-1 topically quick decreases in bacterial levels, faster wound healing, and noticeable improvements in clinical severity scores. Significantly, treatment with phage alone yielded results to those observed with the combination of phage and linezolid across all criteria. These findings suggest that vB_SauM-MUHD-1 has strong potential for treating wounds in this model. While synergy between phages and antibiotics has been noted in other studies (Abedon 2019; Altamirano and Barr 2019). This research was not intended to investigate a mechanistic hypothesis of such synergy. The resulting effects are better described as additive or non-inferior, not synergistic.

Our study demonstrated significant time-dependent differences in both wound healing and bacterial clearance among treatment groups. The superior performance of the phage-treated group and the combination therapy group, reflected by enhanced wound closure and greater bacterial reduction, indicates that these treatment strategies were more effective in controlling infection and promoting tissue repair (Narayanan et al. 2024; Liu et al. 2024). In contrast, the limited improvement observed in the bacterial control group highlights the persistence of infection in the absence of effective therapy. Meanwhile, the phage control group showed no significant change in bacterial load, reflecting the absence of infection rather than a lack of therapeutic efficacy. These findings underscore the importance of selecting appropriate treatment modalities for effective infection control.

The observed association between bacterial reduction and improved wound healing supports the concept that efficient control of bacterial burden is critical for optimal wound recovery. These findings are consistent with previous reports demonstrating that effective antimicrobial interventions accelerate wound healing by reducing microbial load and associated inflammation (Loganathan et al. 2024; Fortaleza et al. 2024). The results emphasize the therapeutic potential of bacteriophage-based therapy particularly when combined with antibiotics in the management of MRSA-infected wounds.

Several constraints and limitations of this study should be noted. The host range was tested against a limited selection of MRSA isolates, and the effectiveness was assessed using only one phage and one bacterial strain in vivo. The current in vivo model used in this study represents an acute murine excisional wound infection with a single MRSA strain, which is suitable for a preclinical proof-of-concept study. The main goal of this model was to assess the therapeutic effectiveness of the bacteriophage and its combination with linezolid under controlled conditions. However, it is important to note that this model may not fully capture the complexity of chronic or biofilm-associated infections. Future research will focus on investigating the efficacy of phage therapy in chronic wound models and against biofilm-forming MRSA strains to better simulate clinical scenarios. Another limitation of the present study is that repeated measurements were obtained from the same animals over time, whereas statistical comparisons were performed using one-way ANOVA at individual time points. Although this approach allows clear between-group comparisons at each stage, it does not account for the longitudinal correlation of repeated observations. A repeated-measures or mixed-effects framework would provide a more comprehensive analysis of temporal changes and should be considered in future studies.

This research shows that vB_SauM-MUHD-1 is a genetically safe, strictly lytic MRSA bacteriophage with helpful biological traits and strong therapeutic effects in a mouse wound infection model. By combining clinical epidemiology, detailed genomic analysis, and in vivo testing, these results support the ongoing development of vB_SauM-MUHD-1 as a topical phage option for treating MRSA wounds. They also contribute to the growing evidence that phage therapy can effectively address antimicrobial-resistant infections.

## Conclusion

This study identifies vB_SauM-MUHD-1 as a genomically safe, strictly lytic MRSA bacteriophage of the *Herelleviridae* (Kayvirus) lineage with favorable biological properties. The phage exhibited efficient replication, a broad operational host range among clinical MRSA isolates, and stability under physiologically relevant conditions. In a murine excisional wound model, topical phage therapy significantly reduced bacterial burden, accelerated wound closure, and improved clinical outcomes compared with untreated infection, performing comparably to phage–linezolid combination therapy and outperforming linezolid monotherapy in bacterial clearance. These findings support further development of vB_SauM-MUHD-1 as a topical phage candidate for MRSA wound infections and warrant evaluation in more complex wound models.

## Supplementary Information

Below is the link to the electronic supplementary material.

Supplementary file1 (PDF 784 kb)

## Data Availability

No datasets were generated or analysed during the current study.
