# Supplementary material for: A new Kayvirus vB_SauM-MUHD-1 combats Methicillin-resistant Staphylococcus aureus wound infections
Source: Appl Microbiol Biotechnol. 2026 Jun 26;110:190. doi: 10.1007/s00253-026-13898-8 (PMC13309502; doi:10.1007/s00253-026-13898-8)
Supplement: Supplementary file 1 [file 253_2026_13898_MOESM1_ESM.pdf]

A new Kayvirus vB_SauM-MUHD-1 combats Methicillin-resistant *Staphylococcus aureus* wound infections

Affiliations: Maha Kabil[1], Noha T. Abou El-Khier[1], Wafaa Mowafy[1], Abeer M. Abd El-Aziz[2], Mohamed Abdelmoteleb[3]*

[1]Department of Medical Microbiology & Immunology, Faculty of Medicine, Mansoura University, Egypt.

[2]Department of Microbiology and Immunology, Faculty of Pharmacy, Mansoura University, Egypt.

[3]Botany Department, Faculty of Science, Mansoura University, Egypt.

***Correspondence:**

Corresponding author: Mohamed Abdelmoteleb

E-mail: mohamed_4abdelmotelb@mans.edu.eg

Keywords: MRSA; wound infection; phage therapy; phage–antibiotic synergy; in vivo murine model.

Supplementary material S1. Bacterial isolates from septic wound samples

Bacterial Strains	Number of Isolates	Percentage (%)
**Gram-positive Cocci (GPC)**	**64**	**53.80%**
- *Staphylococcus aureus*	35	29.40%
- Coagulase-Negative Staphylococci (CoNS)	8	6.70%
- *Streptococcus* spp.	21	17.60%
**Gram-negative Bacilli (GNB)**	**55**	**46.20%**
- *Pseudomonas aeruginosa*	27	22.70%
- *Escherichia coli*	9	7.60%
- *Klebsiella* spp.	12	10.10%
- *Proteus* spp.	7	5.90%
**Total Isolates**	**119**	**100%**

Supplementary material S2. Bacterial isolates from different clinics

Clinical Setting	No. of Patients	*S. aureus*	CoNS	*Strept.* spp.	*P. aeruginosa*	*E. coli*	*Klebsiella* spp.	*Proteus* spp.	Total Isolates
**Diabetic Foot Clinic**	52	18	3	10	15	4	5	3	58
**Surgical Ward**	28	9	2	6	7	2	3	1	30
**Plastic Surgery and Burn Center**	15	4	2	2	4	1	2	1	16
**Surgical Oncology Unit**	12	4	1	3	1	2	2	2	15
**Total**	**107**	**35**	**8**	**21**	**27**	**9**	**12**	**7**	**119**

Supplementary material S3. Comparative antimicrobial resistance profiles of 17 MRSA isolates determined by manual disc diffusion and the VITEK® automated system. The table shows the number and percentage of resistant isolates for each tested antibiotic. ATCC 25923 was used as a quality control strain and remained susceptible to all tested antibiotics.

Antibiotic	Manual Method Resistant (n=17)	Manual Resistance Rate %	VITEK System Resistant (n=17)	VITEK Resistance Rate %	Control ATCC 25923
**Penicillin (PEN)**	17	100	17	100	Susceptible
**Oxacillin (OXA)**	17	100	17	100	Susceptible
**Cefoxitin (FOX)**	17	100	17	100	Susceptible
**Erythromycin (ERY)**	14	82.4	14	82.4	Susceptible
**Clindamycin (CLI)**	14	82.4	14	82.4	Susceptible
**Ciprofloxacin (CIP)**	12	70.6	12	70.6	Susceptible
**Levofloxacin (LEV)**	12	70.6	12	70.6	Susceptible
**Moxifloxacin (MXF)**	–	–	10	58.8	Susceptible
**Gentamicin (GEN)**	9	52.9	9	52.9	Susceptible
**Tetracycline (TET)**	7	41.2	7	41.2	Susceptible
**Minocycline (MIN)**	–	–	3	17.6	Susceptible
**Azithromycin (AZM)**	13	76.5	–	–	Susceptible
**Chloramphenicol (CHL)**	–	–	2	11.8	Susceptible
**Rifampicin (RIF)**	–	–	1	5.9	Susceptible
**Trimethoprim–Sulfamethoxazole (SXT)**	6	35.3	6	35.3	Susceptible
**Amoxicillin–Clavulanate (AMC)**	16	94.1	16	94.1	Susceptible
**Vancomycin (VAN)**	0	0	0	0	Susceptible
**Teicoplanin (TEI)**	–	–	0	0	Susceptible
**Linezolid (LZD)**	0	0	0	0	Susceptible
**Daptomycin (DAP)**	–	–	0	0	Susceptible

Supplementary material S4. Growth characteristics of MRSA-11 hunter strain. Optical density at 600 nm (OD_{600}) and viable counts were used to identify growth phases. The mid-exponential phase (approximately 4.5 h; $OD_{600} \approx 0.60$) was selected for subsequent phage infection experiments.

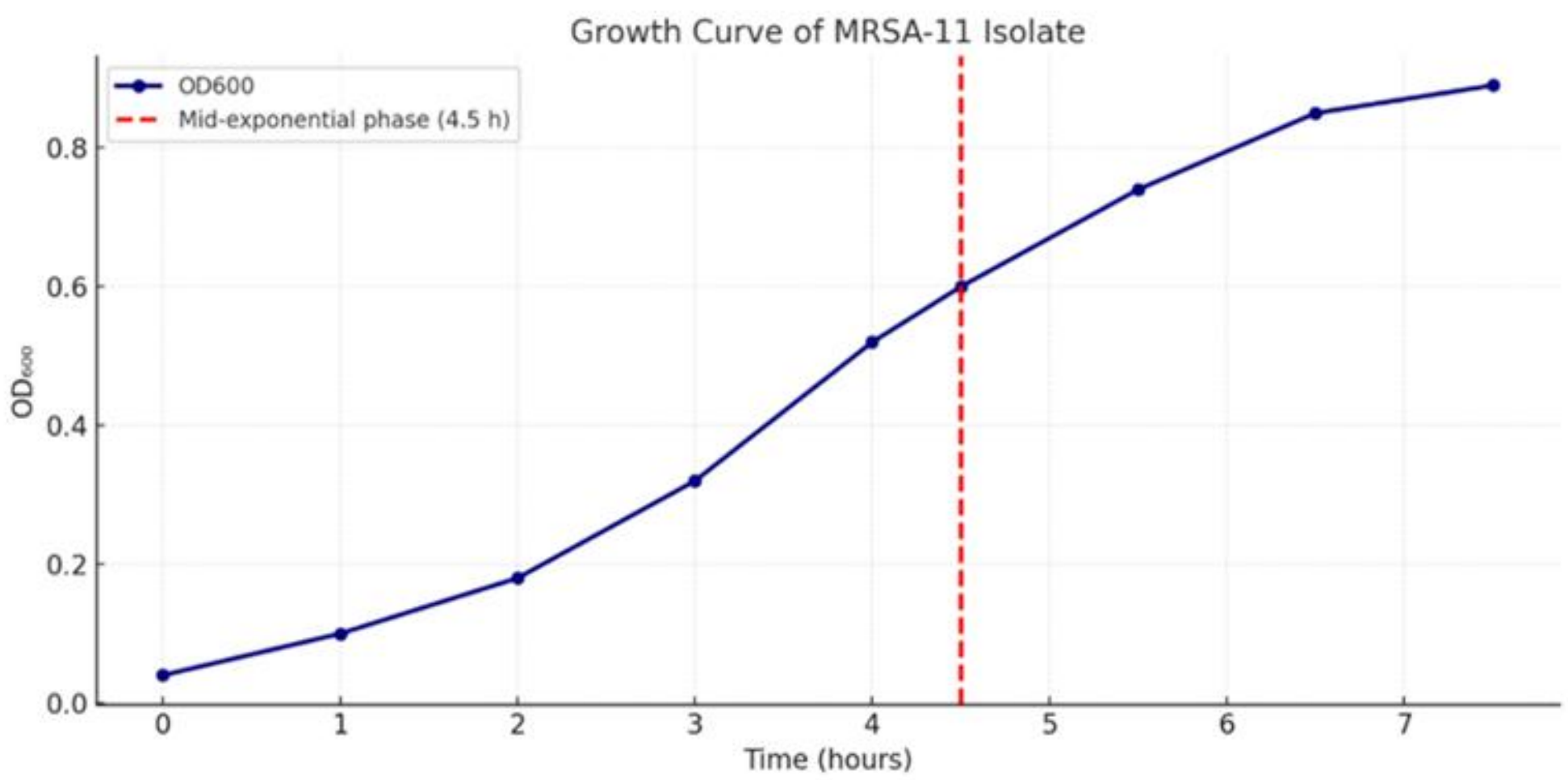

Supplementary material S5. Environmental sampling sites screened for lytic bacteriophages active against hunter MRSA strain. Twelve sewage drainage samples were collected and assessed.

	Sampling Site	Location	Sample Code	Lytic Activity Against MRSA-11	Plaque Formation	Phage Designation
**Healthcare-associated drainage sites**	**Mansoura University Hospital Drainage (MUHD)**	Mansoura University Hospital	MUHD-1	Positive	Yes	vB_SauM-MUHD-1
			MUHD-2	Negative	No	Not detected
			MUHD-3	Negative	No	Not detected
			MUHD-4	Negative	No	Not detected
**Urban drainage sites**	**Al-Mueahada Drainage (AD)**	Talkha City	AD-1	Negative	No	Not detected
			AD-2	Negative	No	Not detected
			AD-3	Negative	No	Not detected
			AD-4	Negative	No	Not detected
	**New Station of Talkha Drainage (NSTD)**	Talkha City	NSTD-1	Negative	No	Not assigned
			NSTD-2	Negative	No	Not detected
			NSTD-3	Positive	Yes	Not detected
			NSTD-4	Negative	No	Not detected
	**Total Samples**	**12**				

Supplementary material S6. Structural and functional annotation of phage vB_SauM-MUHD-1

ORF No.	Strand	Start	Stop	Function	ORF No.	Strand	Start	Stop	Function
ORF1	+	3	1346	hypothetical protein	ORF107	+	67154	67252	hypothetical protein
ORF2	+	1457	1978	hypothetical protein	ORF108	+	67346	67510	hypothetical protein
ORF3	+	1999	5457	hypothetical protein	ORF109	+	67593	67829	hypothetical protein
ORF4	+	5506	5664	hypothetical protein	ORF110	+	67922	68266	hypothetical protein
ORF5	+	5665	7569	hypothetical protein	ORF111	+	68332	68496	hypothetical protein
ORF6	+	7583	7957	hypothetical protein	ORF112	+	68509	68772	hypothetical protein
ORF7	+	7964	9340	capsid and scaffold	ORF113	+	68776	68949	hypothetical protein
ORF8	+	9430	11178	DNA helicase	ORF114	+	68949	69218	hypothetical protein
ORF9	+	11190	12803	hypothetical protein	ORF115	+	69303	69482	hypothetical protein
ORF10	+	12796	14238	DNA helicase	ORF116	-	70109	69819	hypothetical protein
ORF11	+	14317	14736	hypothetical protein	ORF117	-	70448	70212	hypothetical protein
ORF12	+	14736	15761	recombination exonuclease	ORF118	-	70882	70448	hypothetical protein
ORF13	+	15761	16138	hypothetical protein	ORF119	-	71076	70885	hypothetical protein
ORF14	+	16138	18057	recombination related exonuclease	ORF120	-	71321	71073	hypothetical protein
ORF15	+	18057	18653	hypothetical protein	ORF121	-	71803	71321	hypothetical protein
ORF16	+	18668	19735	DNA primase/helicase	ORF122	-	72227	71796	hypothetical protein
ORF17	+	19800	20138	hypothetical protein	ORF123	-	72732	72229	hypothetical protein
ORF18	+	20138	20590	hypothetical protein	ORF124	-	73143	72745	hypothetical protein
ORF19	+	20577	21185	hypothetical protein	ORF125	-	73847	73140	Serine/threonine protein phosphatase
ORF20	+	21203	21595	Ribonucleotide reduction protein NrdI	ORF126	-	75229	74681	hypothetical protein
ORF21	+	21610	23724	Ribonucleotide reductase of class Ib (aerobic), alpha subunit	ORF127	-	75451	75233	hypothetical protein
ORF22	+	23738	24787	Ribonucleotide reductase of class Ib (aerobic), beta subunit	ORF128	-	75646	75452	hypothetical protein
ORF23	+	24805	25134	hypothetical protein	ORF129	-	76373	75636	hypothetical protein
ORF24	+	25118	25438	oxidoreductase	ORF130	-	76540	76436	hypothetical protein
ORF25	+	25646	26242	hypothetical protein	ORF131	-	76791	76552	hypothetical protein
ORF26	+	26252	26557	DNA-binding protein domain	ORF132	-	77182	76793	hypothetical protein
ORF27	+	26633	29851	DNA polymerase I	ORF133	-	77455	77282	hypothetical protein
ORF28	+	29920	30162	hypothetical protein	ORF134	-	77978	77496	hypothetical protein
ORF29	+	30179	30661	hypothetical protein	ORF135	-	78570	78028	hypothetical protein
ORF30	+	30748	32019	hypothetical protein	ORF136	-	79103	78570	hypothetical protein
ORF31	+	32079	33335	recombinase	ORF137	-	79270	79106	hypothetical protein
ORF32	+	33339	33692	hypothetical protein	ORF138	-	79551	79273	hypothetical protein
ORF33	+	33679	34341	hypothetical protein	ORF139	-	80396	79551	hypothetical protein
ORF34	+	34468	35100	hypothetical protein	ORF140	-	81526	80408	hypothetical protein
ORF35	+	35124	35636	major tail protein	ORF141	-	82005	81679	hypothetical protein
ORF36	+	35651	35872	major tail protein	ORF142	-	82414	81998	hypothetical protein
ORF37	+	35969	36229	hypothetical protein	ORF143	-	82849	82547	DNA-binding protein

ORF38	+	36233	36988	hypothetical protein
ORF39	+	36981	38231	hypothetical protein
ORF40	+	38245	38613	hypothetical protein
ORF41	+	38600	38911	hypothetical protein
ORF42	+	38975	39511	hypothetical protein
ORF43	+	39504	40271	hypothetical protein
ORF44	+	40249	40695	hypothetical protein
ORF45	+	40695	41558	hypothetical protein
ORF46	+	41930	42661	hypothetical protein
ORF47	+	42679	43137	hypothetical protein
ORF48	+	43202	43645	hypothetical protein
ORF49	+	43662	44366	hypothetical protein
ORF50	+	44428	44826	hypothetical protein
ORF51	+	44974	45216	hypothetical protein
ORF52	+	45221	45778	hypothetical protein
ORF53	+	45814	45990	hypothetical protein
ORF54	+	45980	46231	hypothetical protein
ORF55	+	46245	46457	hypothetical protein
ORF56	+	46538	47182	hypothetical protein
ORF57	+	47197	47445	hypothetical protein
ORF58	+	47457	47636	hypothetical protein
ORF59	+	47629	47925	hypothetical protein
ORF60	+	47973	48155	hypothetical protein
ORF61	+	48168	48536	hypothetical protein
ORF62	+	48549	48896	hypothetical protein
ORF63	+	48896	49174	hypothetical protein
ORF64	+	49244	49549	hypothetical protein
ORF65	+	49564	49914	hypothetical protein
ORF66	+	49914	50516	hypothetical protein
ORF67	+	50529	50708	hypothetical protein
ORF68	+	50935	51345	hypothetical protein
ORF69	+	51347	51640	hypothetical protein
ORF70	+	51657	51944	hypothetical protein
ORF71	+	51955	52071	hypothetical protein
ORF72	+	52061	52381	hypothetical protein
ORF73	+	52387	53052	hypothetical protein
ORF74	+	53130	53435	hypothetical protein
ORF75	+	53435	53839	hypothetical protein
ORF76	+	53844	54080	hypothetical protein
ORF77	+	54077	54604	Phosphoesterase
ORF78	+	54585	54893	hypothetical protein
ORF79	+	54968	55147	hypothetical protein
ORF80	+	55162	55425	hypothetical protein
ORF81	+	55428	55745	hypothetical protein
ORF82	+	55746	56426	hypothetical protein
ORF83	+	56504	56707	hypothetical protein
ORF84	+	56723	56881	hypothetical protein
ORF85	+	56897	57121	hypothetical protein
ORF86	+	57134	57334	hypothetical protein
ORF87	+	57335	57625	hypothetical protein
ORF88	+	57718	57846	hypothetical protein
ORF89	+	58910	59206	hypothetical protein
ORF90	+	59218	59388	hypothetical protein
ORF91	+	59402	59587	hypothetical protein
ORF92	+	59856	60170	hypothetical protein
ORF93	+	60183	60473	hypothetical protein
ORF94	+	60473	60760	hypothetical protein
ORF95	+	60760	61053	hypothetical protein
ORF96	+	61057	61305	hypothetical protein
ORF97	+	61436	62014	hypothetical protein
ORF98	-	62594	62256	hypothetical protein
ORF99	+	62905	63213	hypothetical protein
ORF100	+	63325	63801	hypothetical protein
ORF101	+	63868	64047	hypothetical protein
ORF102	+	64560	64718	hypothetical protein
ORF103	+	64881	65207	hypothetical protein
ORF104	+	65293	65694	hypothetical protein
ORF105	-	66566	66081	hypothetical protein
ORF106	+	66825	67079	hypothetical protein
ORF144	-	83037	82849	hypothetical protein
ORF145	-	83242	83081	hypothetical protein
ORF146	-	85294	83243	hypothetical protein
ORF147	-	85635	85372	hypothetical protein
ORF148	-	85825	85652	hypothetical protein
ORF149	-	86410	85832	hypothetical protein
ORF150	-	87029	86403	hypothetical protein
ORF151	-	87254	87030	hypothetical protein
ORF152	-	88063	87323	Phosphate starvation-inducible protein (PhoH)
ORF153	-	88729	88115	hypothetical protein
ORF154	-	89170	88745	ribonuclease H
ORF155	-	89351	89160	hypothetical protein
ORF156	-	90015	89374	hypothetical protein
ORF157	-	90235	90005	hypothetical protein
ORF158	-	90465	90238	hypothetical protein
ORF159	-	91268	90576	Lysozyme domain-containing protein
ORF160	-	92259	91465	hypothetical protein
ORF161	-	92567	92259	hypothetical protein
ORF162	-	94168	92681	N-acetylmuramoyl-L-alanine amidase
ORF163	-	94671	94168	holin
ORF164	-	94941	94756	hypothetical protein
ORF165	-	96669	96451	hypothetical protein
ORF166	-	97357	97148	hypothetical protein
ORF167	-	97702	97370	hypothetical protein
ORF168	-	98041	97715	hypothetical protein
ORF169	+	98482	98868	hypothetical protein
ORF170	+	98846	99124	hypothetical protein
ORF171	+	99121	99531	hypothetical protein
ORF172	+	99546	101363	terminase, large subunit
ORF173	+	101356	102177	hypothetical protein
ORF174	+	102164	102337	hypothetical protein
ORF175	+	102334	102813	hypothetical protein
ORF176	+	102855	104066	hypothetical protein
ORF177	+	104130	104492	hypothetical protein
ORF178	+	104510	104881	hypothetical protein
ORF179	+	104885	106576	hypothetical protein
ORF180	+	106771	107544	hypothetical protein
ORF181	+	107563	108519	hypothetical protein
ORF182	+	108635	110026	major capsid protein
ORF183	+	110118	110414	hypothetical protein
ORF184	+	110427	111335	hypothetical protein
ORF185	+	111349	112227	hypothetical protein
ORF186	+	112227	112847	hypothetical protein
ORF187	+	112866	113702	hypothetical protein
ORF188	+	113704	113919	hypothetical protein
ORF189	+	113946	115709	tail sheath
ORF190	+	115782	116210	hypothetical protein
ORF191	+	116307	116447	hypothetical protein
ORF192	+	116493	116783	hypothetical protein
ORF193	+	116804	117262	hypothetical protein
ORF194	+	117275	117469	hypothetical protein
ORF195	+	117486	117638	hypothetical protein
ORF196	+	117706	118017	hypothetical protein
ORF197	+	118149	118604	hypothetical protein
ORF198	+	118648	119184	hypothetical protein
ORF199	+	119240	123295	DNA transfer protein
ORF200	+	123374	125800	Secretory antigen SsaA-like protein
ORF201	+	125814	126701	hypothetical protein
ORF202	+	126701	129247	Glycerophosphoryl diester phosphodiesterase
ORF203	+	129354	130145	hypothetical protein
ORF204	+	130145	130669	hypothetical protein
ORF205	+	130669	131373	hypothetical protein
ORF206	+	131388	132434	baseplate
ORF207	+	132455	133798	hypothetical protein
	+	133674	133800	repeat region
	+	1	127	repeat region
	-	95217	95145	tRNA-Phe-GAA
	-	95297	95224	tRNA-Asp-GTC

Supplementary material S7. Transmembrane topology in **(A)** ORF149 with 7 TMDs and **(B)** ORF177 with 3 TMDs. X-axis: position of amino acid sequences; Y-axis: probability of prediction; Red blocks: transmembrane regions; Blue line: regions external to the membrane; Pink line: regions internal to the membrane.

A

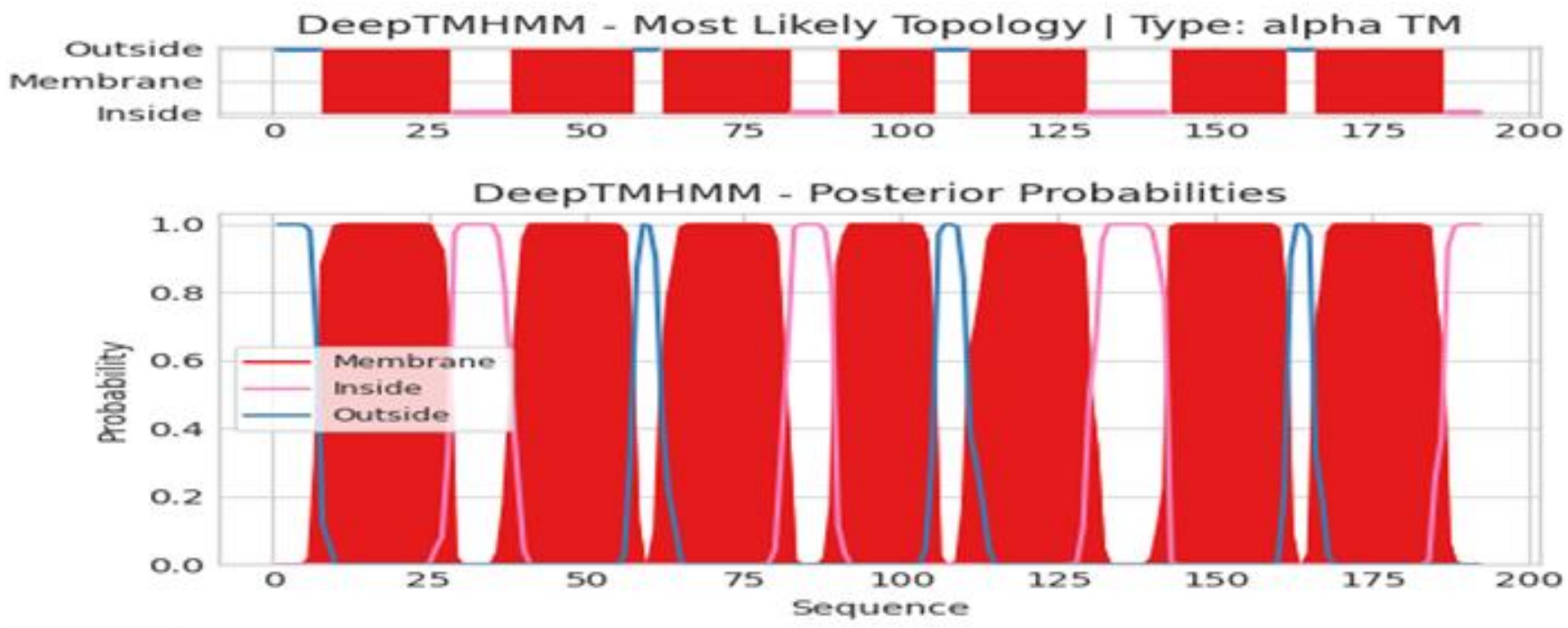

B

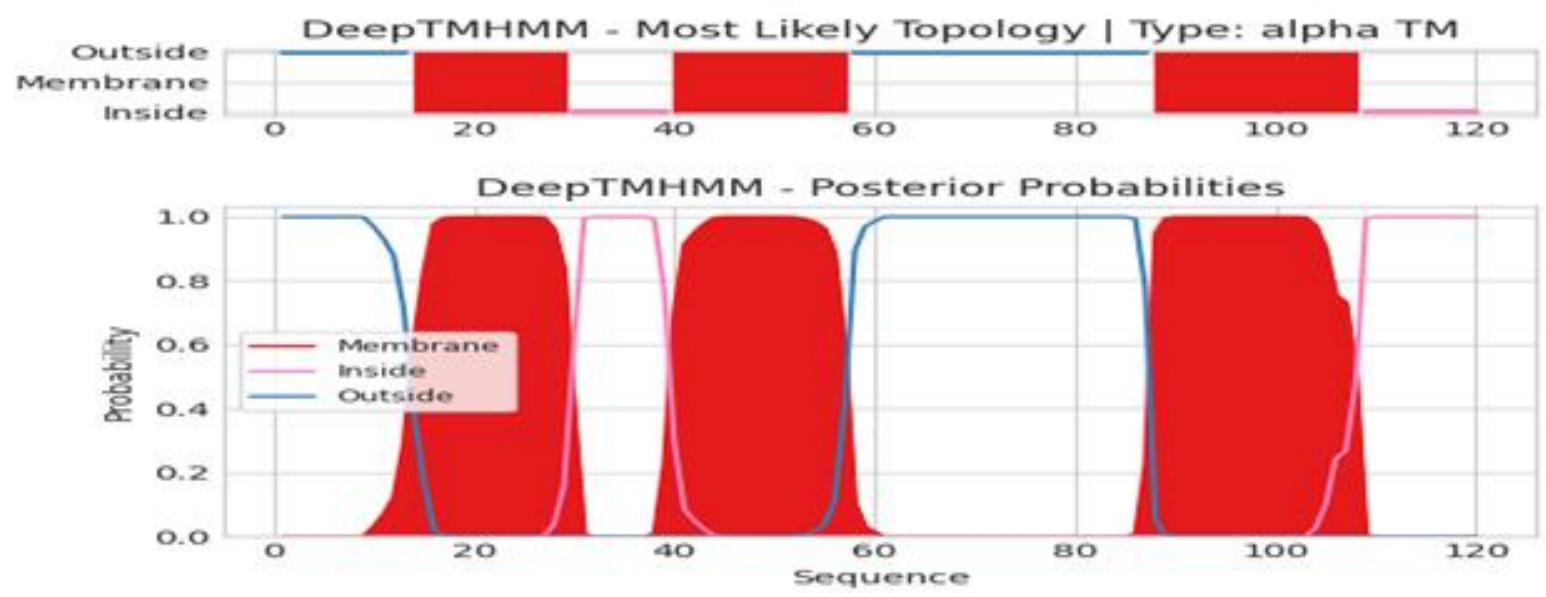

Supplementary material S8. Phylogenetic relationships between the three signature proteins of vB_SauM-MUHD-1 and closely BLASTp matched phages **(A)** PhoH family protein; **(B)** terminase large subunit (TerL); **(C)** major capsid protein.

A

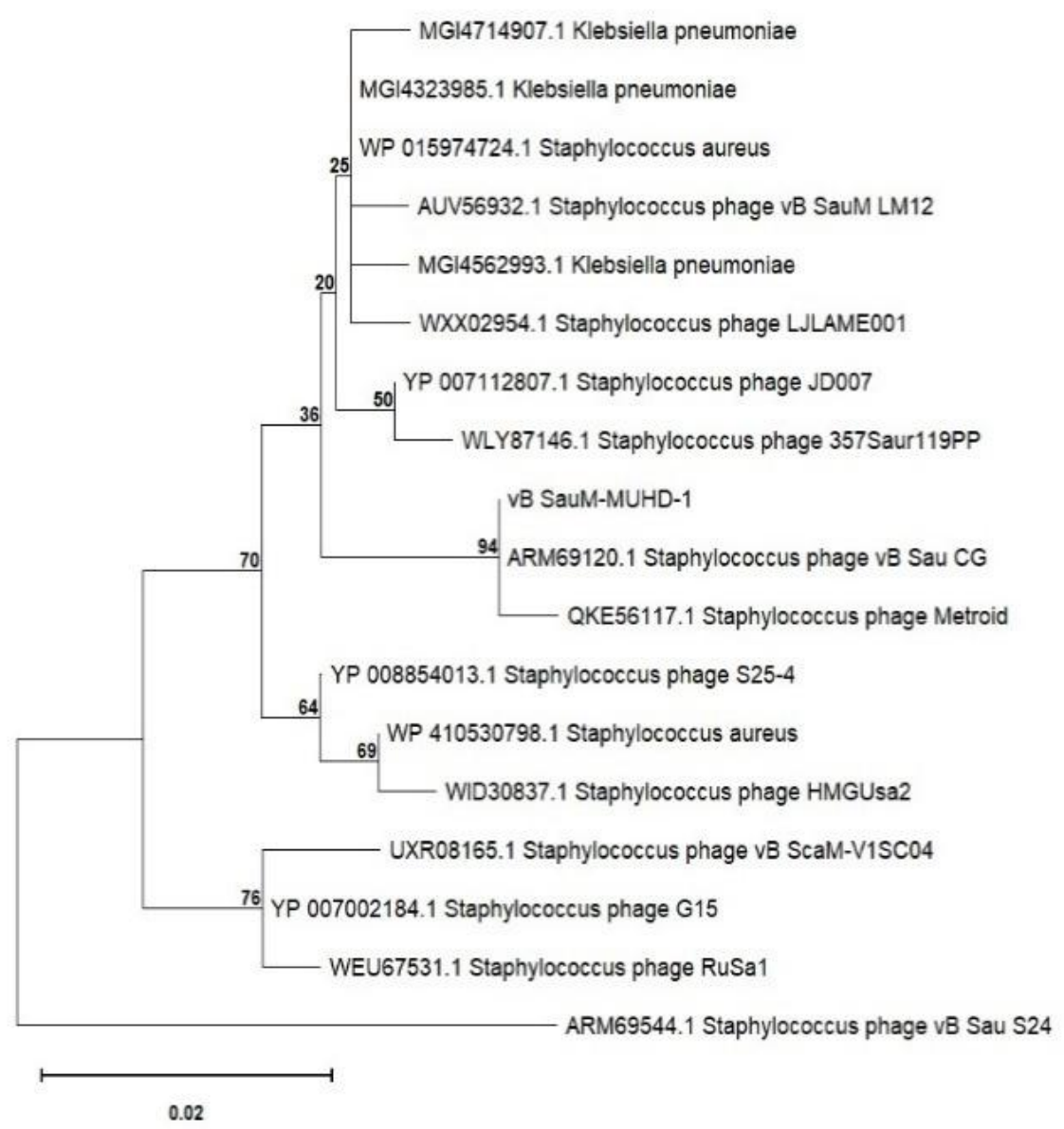

B

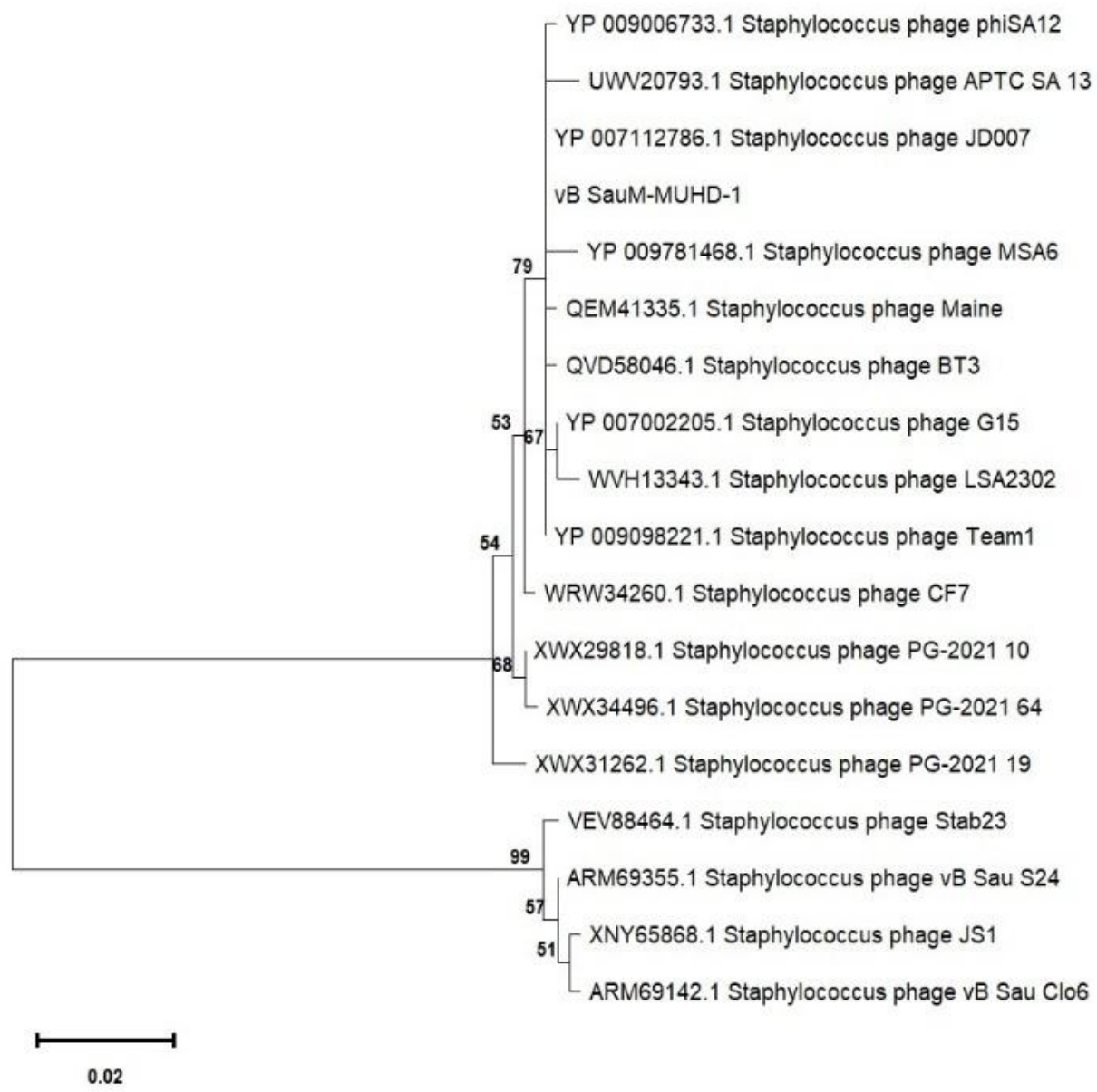

C

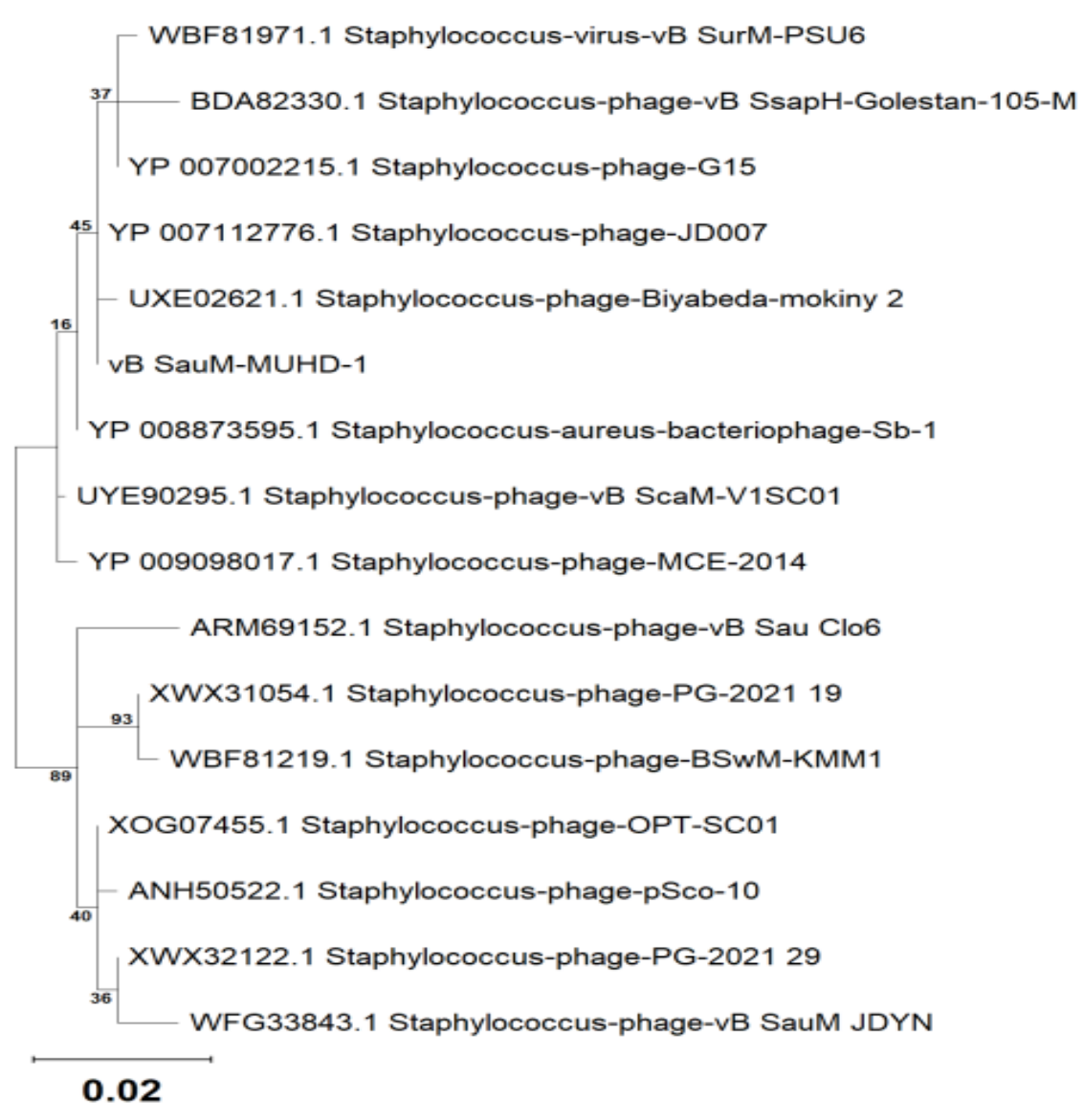

Supplementary material S9. Generalized estimated equation for predicators of wound closure (%)

Wound Closure (%)	Beta	95% CI1	p-value
**Group**			
Group 1	Reference	—	
Group 2	-0.44	-0.44, -0.44	**<0.001***
Group 3	-0.44	-0.44, -0.44	**<0.001***
Group 4	0.80	0.80, 0.80	**<0.001***
Group 5	-0.37	-0.37, -0.37	**<0.001***
Group 6	0.39	0.39, 0.39	**<0.001***
**Time**			
Day 1	Reference	—	
Day 3	21	21, 21	**<0.001***
Day 5	47	47, 47	**<0.001***
Day 7	72	72, 72	**<0.001***
Day 14	92	92, 92	**<0.001***
Day 21	97	97, 97	**<0.001***
**Interaction term (Group* Time)**			
Group 2 * Day 14	-56	-56, -56	**<0.001***
Group 3 * Day 14	-11	-11, -11	**<0.001***
Group 4 * Day 14	-2.9	-2.9, -2.9	**<0.001***
Group 5 * Day 14	-5.5	-5.5, -5.5	**<0.001***
Group 6 * Day 14	1.2	1.2, 1.2	**<0.001***
Group 2 * Day 21	-53	-53, -53	**<0.001***
Group 3 * Day 21	-4.2	-4.2, -4.2	**<0.001***
Group 4 * Day 21	-1.7	-1.7, -1.7	**<0.001***
Group 5 * Day 21	-1.7	-1.7, -1.7	**<0.001***
Group 6 * Day 21	-1.1	-1.1, -1.1	**<0.001***
Group 2 * Day 3	-16	-16, -16	**<0.001***
Group 3 * Day 3	-0.73	-0.73, -0.73	**<0.001***
Group 4 * Day 3	6.1	6.1, 6.1	**<0.001***
Group 5 * Day 3	1.7	1.7, 1.7	**<0.001***
Group 6 * Day 3	7.7	7.7, 7.7	**<0.001***
Group 2 * Day 5	-38	-38, -38	**<0.001***
Group 3 * Day 5	-4.7	-4.7, -4.7	**<0.001***
Group 4 * Day 5	7.1	7.1, 7.1	**<0.001***
Group 5 * Day 5	2.7	2.7, 2.7	**<0.001***
Group 6 * Day 5	13	13, 13	**<0.001***
Group 2 * Day 7	-51	-51, -51	**<0.001***
Group 3 * Day 7	-12	-12, -12	**<0.001***
Group 4 * Day 7	-0.14	-0.14, -0.14	**<0.001***
Group 5 * Day 7	-5.5	-5.5, -5.5	**<0.001***
Group 6 * Day 7	7.8	7.8, 7.8	**<0.001***

CI: Confidence interval

Supplementary material S10. Generalized estimated model for predicators of bacterial load

Bacterial load	Beta	95% CI1	p-value
**Group**			
Group 1	Reference	—	
Group 2	7.7	7.7, 7.7	**<0.001***
Group 3	0.00	0.00, 0.00	0.9
Group 4	3.9	3.9, 3.9	**<0.001***
Group 5	4.9	4.9, 4.9	**<0.001***
Group 6	3.6	3.6, 3.6	**<0.001***
**Time**			
Day 1	Reference	—	
Day 3	0.00	0.00, 0.00	0.9
Day 5	0.00	0.00, 0.00	0.9
Day 7	0.00	0.00, 0.00	0.9
Day 14	0.00		
Day 21	0.00	0.00, 0.00	0.9
**Interaction term (Group * Time)**			
Group 2 * Day 14	-0.25	-0.25, -0.25	**<0.001***
Group 3 * Day 14	0.00	0.00, 0.00	0.9
Group 4 * Day 14	-1.2	-1.2, -1.2	**<0.001***
Group 5 * Day 14	-1.0	-1.0, -1.0	**<0.001***
Group 6 * Day 14	-1.2	-1.2, -1.2	**<0.001***
Group 2 * Day 21	-0.38	-0.38, -0.38	**<0.001***
Group 3 * Day 21	0.00		
Group 4 * Day 21	-1.1	-1.1, -1.1	**<0.001***
Group 5 * Day 21	-0.90	-0.90, -0.90	**<0.001***
Group 6 * Day 21	-1.2	-1.2, -1.2	**<0.001***
Group 2 * Day 3	-0.09	-0.09, -0.09	**<0.001***
Group 3 * Day 3	0.00	0.00, 0.00	0.9
Group 4 * Day 3	-0.36	-0.36, -0.36	**<0.001***
Group 5 * Day 3	-0.31	-0.31, -0.31	**<0.001***
Group 6 * Day 3	-0.36	-0.36, -0.36	**<0.001***
Group 2 * Day 5	-0.12	-0.12, -0.12	**<0.001***
Group 3 * Day 5	0.00		
Group 4 * Day 5	-0.85	-0.85, -0.85	**<0.001***
Group 5 * Day 5	-0.67	-0.67, -0.67	**<0.001***
Group 6 * Day 5	-0.85	-0.85, -0.85	**<0.001***
Group 2 * Day 7	-0.12	-0.12, -0.12	**<0.001***
Group 3 * Day 7	0.00		
Group 4 * Day 7	-1.1	-1.1, -1.1	**<0.001***
Group 5 * Day 7	-0.89	-0.89, -0.89	**<0.001***
Group 6 * Day 7	-1.1	-1.1, -1.1	**<0.001***

CI: Confidence interval
